# Identification of asymptomatic *Leishmania* infections: a scoping review

**DOI:** 10.1186/s13071-021-05129-y

**Published:** 2022-01-05

**Authors:** Ana Victoria Ibarra-Meneses, Audrey Corbeil, Victoria Wagner, Chukwuemeka Onwuchekwa, Christopher Fernandez-Prada

**Affiliations:** 1 Département de Pathologie et Microbiologie, Faculté de Médecine Vétérinaire, Université de Montréal, Saint-Hyacinthe, QC Canada; 2 The Research Group on Infectious Diseases in Production Animals (GREMIP), Faculty of Veterinary Medicine, Université de Montréal, Saint Hyacinthe, Canada; 3 Barcelona Institute for Global Health (ISGlobal), Hospital Clínic, University of Barcelona, Barcelona, Spain

**Keywords:** *Leishmania*, Leishmaniasis, Asymptomatic, Blood donor, Molecular test, Serological test, Cellular test

## Abstract

**Background:**

Asymptomatic *Leishmania* infection may play an important role in the transmission of the parasite in endemic areas. At present there is no consensus on the definition of asymptomatic *Leishmania* infection, nor is there a safe and accessible gold standard test for its identification.

**Methods:**

This paper presents a scoping review to summarize definitions of asymptomatic *Leishmania* infection found in the literature, as well as to detail the approach (molecular, serological, cellular, and/or parasitological tests) used by researchers to identify this asymptomatic population. A scoping review of published and gray literature related to asymptomatic *Leishmania* infection was conducted; retrieved citations were screened based on predefined eligibility criteria, and relevant data items were extracted from eligible articles. The analysis is descriptive and is presented using tables, figures, and thematic narrative synthesis.

**Results:**

We conducted a screening of 3008 articles, of which 175 were selected for the full review. Of these articles, we selected 106 that met the inclusion criteria. These articles were published between 1991 and 2021, and in the last 5 years, up to 38 articles were reported. Most of the studies were conducted in Brazil (26%), Spain (14%), India (12%), Bangladesh (10%), and Ethiopia (7%). Of the studies, 84.9% were conducted in the immunocompetent population, while 15.1% were conducted in the immunosuppressed population (HIV, immunosuppressive drugs, and organ transplantation population). We report 14 different techniques and 10 strategies employed by researchers to define asymptomatic *Leishmania* infection in an endemic area.

**Conclusions:**

The definition of asymptomatic *Leishmania* infection is not unified across the literature, but often includes the following criteria: residence (or extended stay) in a *Leishmania*-endemic area, no reported signs/symptoms compatible with leishmaniasis, and positive on a combination of serological, molecular, cellular, and/or parasitological tests. Caution is recommended when comparing results of different studies on the subject of asymptomatic infections, as the reported prevalence cannot be confidently compared between areas due to the wide variety of tests employed by research groups. More research on the importance of asymptomatic immunosuppressed and immunocompetent *Leishmania*-positive populations in leishmaniasis epidemiology is required.

**Graphical Abstract:**

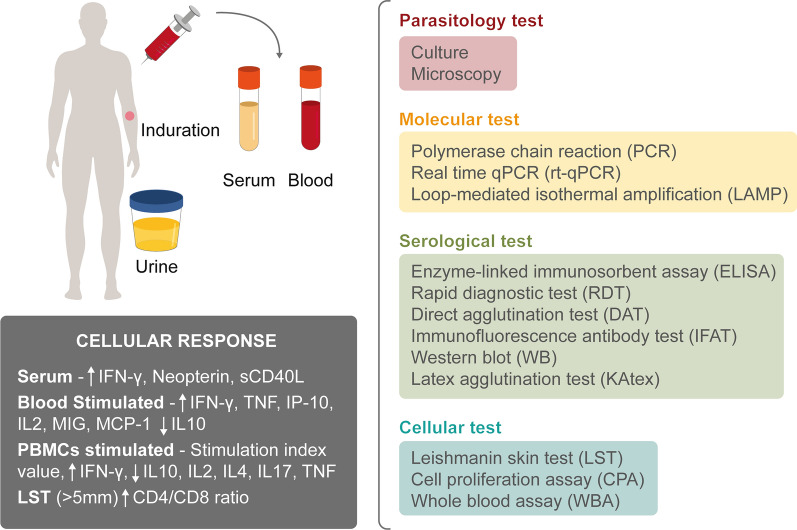

**Supplementary Information:**

The online version contains supplementary material available at 10.1186/s13071-021-05129-y.

## Background

Leishmaniasis is considered a neglected tropical disease (NTD). It is a vector-borne infectious disease caused by parasites of the genus *Leishmania*, transmitted by the bite of infected female sand flies [1, 2]. An estimated 12 million cases of leishmaniasis exist worldwide, with 350 million people at risk of infection [3]. Cutaneous leishmaniasis (CL) and visceral leishmaniasis (VL) are the most severe clinical forms of the disease; CL affects the skin, while VL affects the internal organs of the infected patient [4]. The evolution of the disease occurs progressively over a period of weeks or even months and is influenced by environmental, parasite-, and host-related factors [5]. VL is fatal in 95% of cases if left untreated [2].

Leishmaniasis is endemic in 98 countries; however, official data underestimate the reality of human leishmaniasis due to the low number of mandatory reporter countries (32/98), the large number of cases that are incorrectly diagnosed, official data being obtained exclusively from passive case detection, and the large, unreported asymptomatic population [3, 6].

Asymptomatic infection represents approximately 20–60% of *Leishmania* spp. infection in endemic areas [7, 8]. Although the asymptomatic population likely represents the highest proportion of infection, there is no agreed definition of the condition or accurate means by which to detect a subject with asymptomatic *Leishmania* infection [9]. Some authors define a subject with asymptomatic infection as a healthy individual living in an endemic area who tests positive on a molecular [polymerase chain reaction (PCR), quantitative PCR (qPCR), or loop-mediated isothermal amplification (LAMP)], serological [direct agglutination test (DAT), enzyme-Linked immunosorbent assay (ELISA) test, rK39-immunochromatographic rapid test (rK39-RDT), immunofluorescence antibody test (IFAT), or Western blot (WB)], or cellular test [leishmanin skin test (LST), interferon gamma release assay (IGRA), whole blood assay (WBA), cell proliferation assay (CPA)]; while others consider a combination of the above tests [10–14].

The asymptomatic population is of vital importance for several reasons. Firstly, they may well serve as a reservoir of parasites, presenting a risk to public health through infection of the phlebotomine vector [15]. Immunosuppression is one of the risk factors that can increase progression to clinical manifestation in asymptomatic subjects. Human immunodeficiency virus (HIV) infection, immunosuppressive drugs, and organ transplantation are the most widely studied risk factors for co-infection with *Leishmania*. HIV infection increases the risk of developing VL by 100–2320 times, while the risk is increased by 20–100 times after treatment with immunosuppressive drugs and after organ transplantation [16, 17]. As such, special attention should be paid to this asymptomatic immunosuppressed (IS) population in endemic areas.

Further interest in the asymptomatic population stems from the mystery surrounding leishmaniasis disease progression. It is well known that a large proportion of those infected with *Leishmania* spp. never demonstrate clinical manifestations of the disease [8]. It has been suggested that progression towards symptomatic VL likely results from a combination of various host, parasite, and sociodemographic factors [18]. A clearer understanding of the manifold factors leading to the development of clinical leishmaniasis could inform the treatment of asymptomatic patients to improve disease outcome, as well as reduce parasite transmission from this potentially significant reservoir [15].

For these myriad reasons, there is an urgent need to establish a specific definition of “asymptomatic *Leishmania* infection,” such that future studies may contribute to the development of new leishmaniasis control strategies through standardized and methodical means. To this end, our aims in this study are to outline current approaches used to describe asymptomatic *Leishmania* infection in endemic areas and map out approaches previously used for the study of asymptomatic *Leishmania* infection in blood banks, epidemiological surveys, and through screening of patients in endemic areas. Furthermore, frequently employed definitions of “asymptomatic *Leishmania* infection” and their associated diagnostic tests are discussed, such that we may suggest common usage guidelines concerning these topics to inform future studies in the field.

## Methods

### Protocol and registration

We developed the protocol for the scoping review in line with the Preferred Reporting Items for Systematic Review and Meta-Analysis Protocols (PRISMA-P). The protocol was developed before beginning the search and was reviewed and approved by all members of the review team.

### Eligibility criteria

For this scoping review, we sought to identify primary studies reporting on asymptomatic *Leishmania* infection within human populations in an endemic area (see Additional file 1: Table S1 for the list of endemic countries). Eligible studies could include populations of any age, sex, and health status. Only studies in which a diagnostic technique was employed to identify *Leishmania* in asymptomatic people were considered eligible. We included articles in English, French, Spanish, and Portuguese, and from any period. The eligible study designs included surveillance studies, cross-sectional studies, cohort and case–control studies, and interventional studies. Articles were excluded if they reported on studies of symptomatic *Leishmania* infection, involved only animal populations, or did not include a diagnostic technique. Studies not based on primary data, such as reviews and modelling studies, were also ineligible for this scoping review.

For our study, we considered subjects as asymptomatically infected with *Leishmania* if they met the following criteria: no signs/symptoms of leishmaniasis (based on clinical examination by a medical professional and/or medical history as declared by the patient), positive on at least one diagnostic test (serological, molecular, cellular, and/or parasitological), and residence (or history of extended stay) in an area of leishmaniasis endemicity.

### Information sources

To identify potentially relevant articles, we conducted a detailed search of the PubMed, Web of Science, and LILACS databases. All three bibliographic sources were searched on 11 August 2021. We also conducted a manual search of eligible articles for potentially relevant articles that may have been missed in the bibliographic search.

### Search

Our search strategy combined the broad terms “*Leishmania*” and “asymptomatic.” We included alternate terms within each concept to improve the sensitivity of the search. No restrictions on language or publication period were included in the search. The initial search strategy was developed by AVIM, CO, and AC, and was reviewed by CFP. The final search strategy was modified via an iterative and consultative process involving all members of the review team. For this scoping review, we did not include gray literature.

The complete search syntax for the PubMed search is presented below, combining Medical Subject Headings (MeSH) and free-text search (Additional file 2: Table S2).

((*Leishmania*[*MeSH Terms*])* OR *(leishmania*))* AND *(((asymptomatic*)* OR *(*carrier*)* OR *(*blood donor*)* OR *(*subclinical*)))

### Selection of sources of evidence

The citations returned from the electronic database searches were imported into EndNote, where duplicate records were identified and deleted. The de-duplicated citations were subsequently imported into COVIDENCE, where further de-duplication was carried out and articles were assessed for eligibility.

AVIM and AC independently screened the titles and abstracts of records to identify potentially relevant studies. Subsequently, AVIM, AC and VW independently read the full text of the potentially relevant articles and selected those that met the eligibility criteria. At each stage of the selection process, two reviewers had to independently agree on the assignment of each article. The agreement was high between voting members, with 95% agreement at the title and abstract stage, and 81–88% at the full-text stage. Disagreement in voting at each stage was resolved by CFP following consultation with the voting pair.

### Data charting process

Predefined data items were identified by the review team through a consultative process during the planning of the scoping review and subsequently integrated into an extraction form. The data extraction form was designed and piloted in Excel using some eligible papers and modified as required. One member of the team (AVIM, AC, and VW) conducted the initial data extraction, and a second member crosschecked the extracted data to ensure completeness and accuracy (AVIM, AC, and VW). Discordance in extracted data was resolved by consensus between the reviewers.

### Data items

In Table 1 below, we present details of the data items collected as part of the scoping review.

**Table 1:** Data items and characterization

Data item	Characterization
Author	Last name of first author
Year	Year of publication
WHO region	WHO region where study was conducted: European Region, Americas, Eastern Mediterranean Region, South-East Asia, African Region
Country	The country or countries where the study was conducted
Objective of study	As outlined by the authors. This was extracted verbatim and subsequently thematically characterized into analyzable data items, e.g., prevalence survey, test validation, etc.
Population description	Description of the study population, including the methods of selection, e.g., household contacts, blood donors and volunteers
Population size	The size of the sampled population
*Leishmania* manifestation	The clinical manifestation of *Leishmania* applied to determine symptomatology, including visceral (VL), cutaneous (CL), and post-kala-azar dermal leishmaniasis (PKDL)
Clinical status	The overall clinical status of the study population, including: immunocompetent (IC), immunosuppressed (IS), HIV-infected (HIV), and solid organ transplant (SOT)
History of clinical *Leishmania* disease	Whether a history of previous leishmaniasis was confirmed in the study population
*Leishmania* species	The species of *Leishmania* under investigation. These include *L. donovani, L. infantum*, *L. major*, *L. chagasi*, *L. braziliensis*, *L. amazonensis*, *L. mexicana*, *L. guyanensis*, *L. panamensis*
Diagnostic test used	The diagnostic test used by the investigators, as reported in the manuscript
Definition of asymptomatic disease	As reported directly by the authors where available; alternatively, an implicit definition was inferred from information available in the manuscript

### Outcomes and prioritization

The outcome of interest in our review is a description of the common definitions of “asymptomatic *Leishmania* infection” found in the literature, as well as the techniques used for the detection of this population in various endemic areas. No other outcomes are considered for prioritization**.**

### Critical appraisal of individual sources of evidence

We did not conduct a formal critical appraisal of included articles as part of this scoping review, as the aim of this review was to describe the scope of research and not to present pooled study results.

### Synthesis of results

We used a descriptive approach in the synthesis and presentation of the scoping review findings. We present an overview of the study selection process through narrative and PRISMA flowchart. A summary of the included articles by year of publication, study country, study setting, study aim(s), and population characteristics is presented in a descriptive table. We subsequently describe how researchers had operationalized and defined asymptomatic *Leishmania* infection and summarize these strategies using a thematic approach. We used a combination of deductive and inductive processes to obtain the definitions based on explicit or implicit case definitions obtained from included articles. We further identified test–test comparison pairs based on a reduced number of eligible articles where two or more tests were used in parallel. *Leishmania* microscopy and culture were collectively classified as “parasitology tests,” while WBA, LST and CPA tests were categorized as “cellular immunology tests.” The other tests included in this study—serological and molecular—were considered individually. Using the suite of network commands in Stata [16], we constructed a network showing comparisons made between tests among eligible studies. We inspected linkages (or interconnectivity) between test types in the network to identify potential gaps in test comparison. Using a combination of narrative synthesis, graphs, and network diagrams, we demonstrate how specific tests were used independently or in combination in the included studies.

## Results

### Searching for an asymptomatic definition

**Fig. 1:**
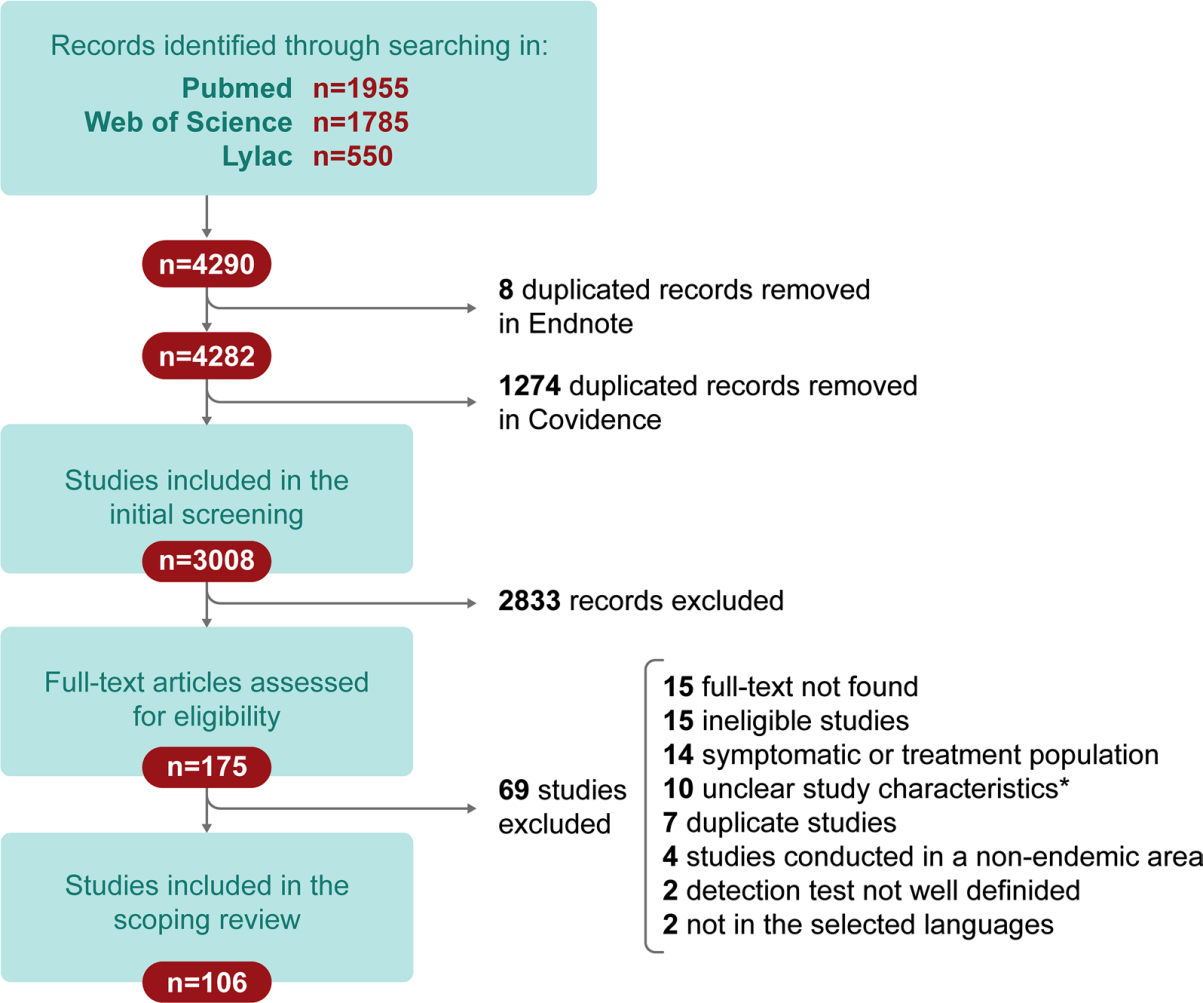
Flow chart of literature search and screening. *Unclear study characteristics: indicates works where study population inclusion/exclusion criteria, diagnostic methods, and/or overall conceptualization was ambiguous

The diagram used in this scoping review for the selection of articles is shown in Fig. 1, following the Tricco et al. guideline [19]. Initially, the first search in the three chosen databases yielded 4290 articles. After eliminating duplicates, 3008 articles were selected for screening by title and abstract. After the first screening, 175 articles were retrieved. Of these 175 articles, 69 were discarded because they did not meet the inclusion criteria described above. Thus, in total, we selected 106 articles for this scoping review.

**Fig. 2:**
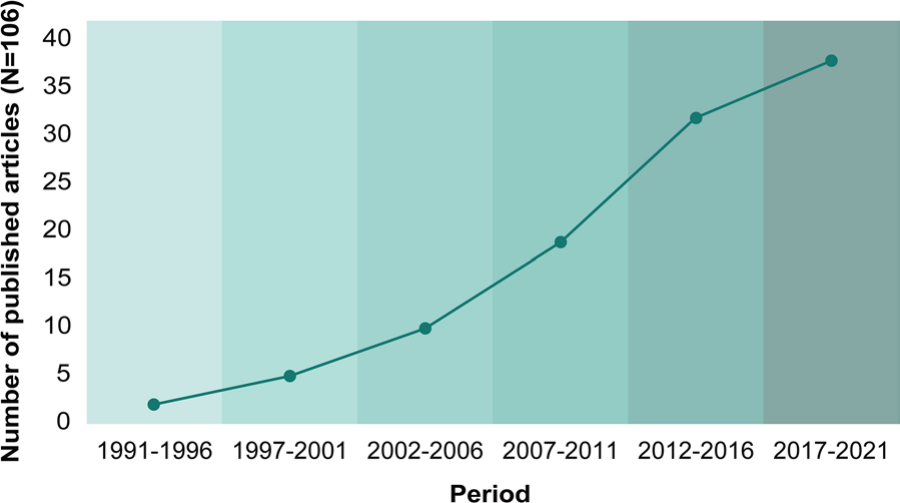
Yearly frequency of studies published on asymptomatic *Leishmania* infection

The number of studies on the identification of the asymptomatic *Leishmania* populations in endemic areas has increased over the years. Figure 2 shows an exponential increase in the number of studies carried out in 5-year periods. Between 1991 and 1996, only two studies were published describing asymptomatic *Leishmania* infection, while in the current period (2017–2021), as of August 2021, 38 articles had been published.

**Fig. 3:**
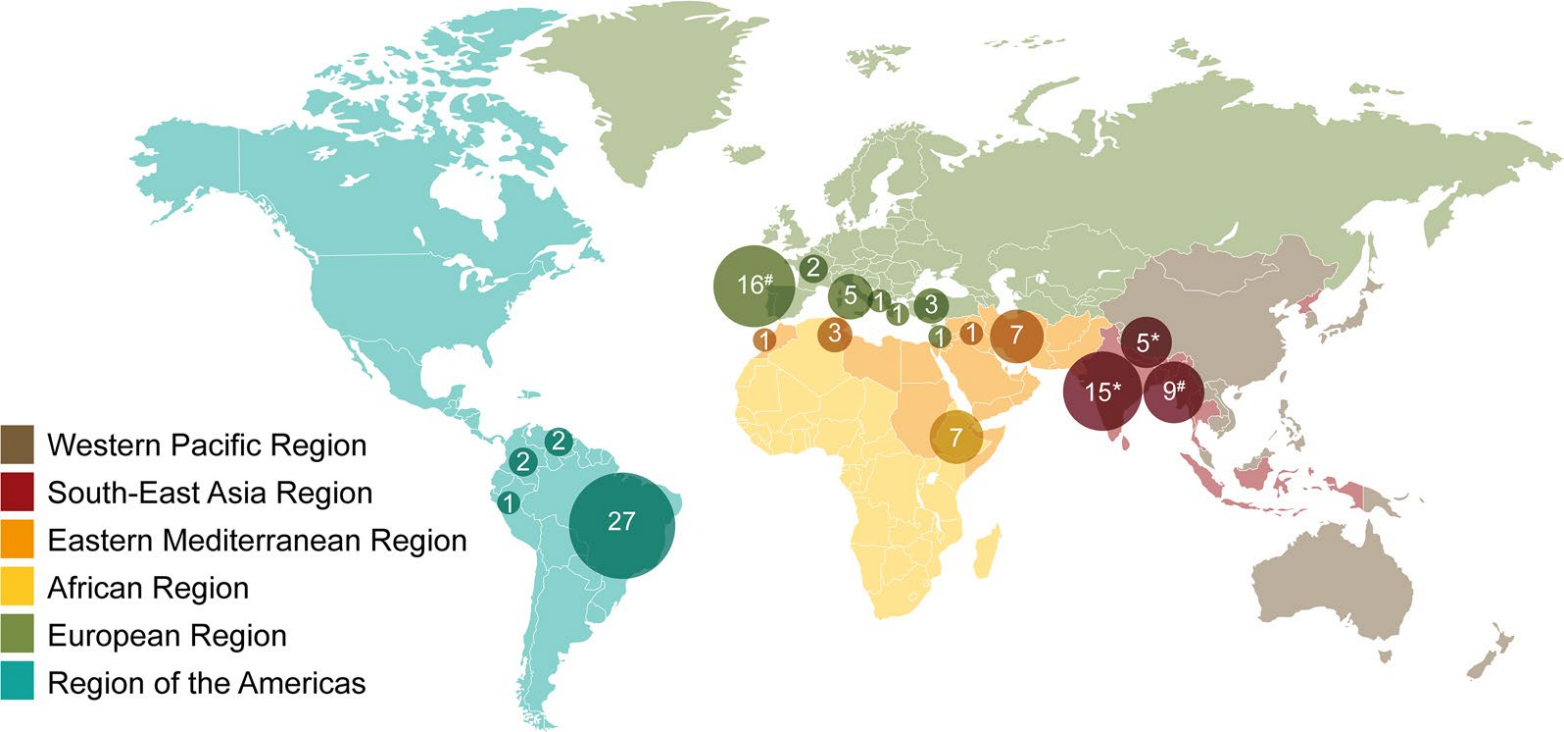
Distribution by WHO geographic region and countries of studies included in the scoping review. *#*A study co-conducted in Spain and Bangladesh; *a study co-conducted in India and Nepal

Articles from 19 countries were identified for this study. Of the 106 total articles, seven studies were conducted in only one country in the World Health Organization (WHO) African region (Ethiopia). Meanwhile, in the WHO South-East Asia Region, 26 studies from three countries (Bangladesh, India, Nepal) were included. In the WHO Eastern Mediterranean Region, 12 studies were included from four countries (Iran, Iraq, Morocco, Tunisia), while in the WHO European Region, 29 studies were included from seven countries (Croatia, France, Greece, Israel, Italy, Spain, Turkey) (Fig. 3). Of the total number of studies, 26% (27/106) were conducted in Brazil, followed by Spain (14%; 15/106), India (12%; 13/106), Bangladesh (8%; 8/106), Ethiopia (7%; 7/106), Iran (7%; 7/106) and Italy (5%; 5/106).

### Description of the asymptomatic studies included

Information from the 106 studies describing asymptomatic *Leishmania* infection in endemic areas is described in Table 2. All the studies included were primary studies, where subjects did not manifest any symptoms or signs of the disease. The age of subjects ranged from 2 years to  > 60 years. Among studies, 94.3% were associated with asymptomatic infection in endemic areas of *L. infantum* and/or *L. donovani*, while 5.7% of studies were performed in the endemic area of *L. major*, *L. braziliensis*, *L. panamensis*, *L. amazonensis*, *L. mexicana*, and *L. guyanensis*.

**Table 2:** Description of the 106 studies included, divided by WHO regions

Authors	Year	Country	Size (*n*)	Type of leishmaniasis	Clinical manifestation	VL history	Study population	Species	Objective	Ref.
WHO African Region										
Bejano et al.	2021	Ethiopia	1342	VL	IC	nd	Households	*L. donovani*	Prevalence	[20]
									Epidemiology	
									Risk factors	
Tadese et al.	2019	Ethiopia	1099	VL	IC	None	Volunteers	*L. donovani*	Prevalence	[21]
Ayehu et al.	2018	Ethiopia	185	VL	IC	None	Laborers	*L. donovani*	Prevalence	[22]
									Risk factors	
Custodio et al.	2012	Ethiopia	639	VL	IC	None	Households	*L. donovani*	Risk factors	[23]
									Epidemiology	
Gadisa et al.	2012	Ethiopia	605	VL	IC	None	Households	*L. donovani*	Test evaluation	[24]
Griensven et al.	2019	Ethiopia	511	VL	HIV	None	Volunteers	*L. donovani*	Prevalence	[13]
									Incidence	
									Disease progression	
Adriaensen et al.	2018	Ethiopia	35	VL	HIV	Yes	Volunteers	*L. donovani*	Immunological biomarkers	[25]
									Test evaluation	
WHO South-East Asia Region										
Basnyat et al.	2021	Nepal	189	VL	IC	None	Households	*L. donovani*	Prevalence	[26]
									Leishmaniasis contacts	
									Epidemiology	
Cloots et al.	2021	India	94	VL	IC	None	Volunteers	*L. donovani*	Test evaluation	[27]
Owen et al.	2021	Bangladesh	720	VL	IC	None	Households	*L. donovani*	Test evaluation	[28]
									Leishmaniasis contacts	
Johanson et al.	2020	India	109	VL	IC	None	Households	*L. donovani*	Prevalence	[29]
									Leishmaniasis contacts	
									Epidemiology	
Chakravarty et al.	2019	India	1606	VL	IC	nd	Households	*L. donovani*	Test evaluation	[18]
									Disease progression	
Mondal et al.	2019	Bangladesh	200	VL	IC	None	Volunteers	*L. donovani*	Immunological biomarkers	[30]
									Disease progression	
									Test evaluation	
Singh et al.	2018	India	64	VL	IC	nd	Volunteers	*L. donovani*	Immunological biomarkers	[31]
Kaushal et al.	2017	India	246	VL	IC	Yes	Volunteers	*L. donovani*	Prevalence	[32]
Saha et al.	2017	India	2603	VL	IC	None	Volunteers	*L. donovani*	Prevalence	[33]
									Disease progression	
Banu et al.	2016	Bangladesh, Australia	706	VL	IC	None	Blood donors and volunteers	*L. donovani*	Test evaluation	[34]
Banu et al.	2016	Bangladesh	257	VL	IC	None	Households	*L. donovani*	Prevalence	[35]
									Leishmaniasis contacts	
Das et al.	2016	India	5144	VL and PKDL	IC	None	Households	*L. donovani*	Leishmaniasis contacts	[36]
									Disease progression	
									Epidemiology	
Timilsina et al.	2016	Nepal	507	VL	IC	None	Blood donors	*L. donovani*	Prevalence	[37]
Vallur et al.	2016	Bangladesh	104	VL	IC	None	Households	*L. donovani*	Test evaluation	[38]
Picado et al.	2014	India and Nepal	510	VL	IC	None	Households	*L. donovani*	Risk factors	[39]
									Disease progression	
									Epidemiology	
Sudarshan et al.	2014	India	130	VL	IC	nd	Households	*L. donovani*	Test evaluation	[40]
									Disease progression	
Sudarshan et al.	2014	India	1469	VL	IC	nd	Households	*L. donovani*	Test evaluation	[41]
									Disease progression	
Huda et al.	2013	Bangladesh	1195	VL	IC	None	Blood donors	*L. donovani*	Prevalence	[42]
Srivastava et al.	2013	India	286	VL	IC	None	Households	*L. donovani*	Prevalence	[43]
									Test evaluation	
Ostyn et al.	2011	India and Nepal	9034	VL	IC	None	Volunteers	*L. donovani*	Disease progression	[44]
Topno et al.	2010	India	335	VL	IC	Yes	Households	*L. donovani*	Prevalence	[45]
									Disease progression	
Bhattarai et al.	2009	Nepal	231	PKDL	IC		Households	*L. donovani*	Test evaluation	[46]
Gidwani et al.	2009	India	870	VL	IC	None	Households	*L. donovani*	Prevalence	[47]
									Leishmaniasis contacts	
									Disease progression	
									Epidemiology	
Sinha et al.	2008	India	172	VL	IC	None	Households	*L. donovani*	Test evaluation	[48]
									Leishmaniasis contacts	
Bern et al.	2007	Bangladesh	1379	VL	IC	None	Households	*L. donovani*	Incidence	[49]
									Risk factors	
Chowdhury et al.	1993	Bangladesh	17 826	VL	IC	nd	Households	*L. donovani*	Test evaluation	[50]
									Prevalence	
WHO Eastern Mediterranean Region										
Mody et al.	2019	Iraq	200	VL	IC	Yes	Soldiers	*L. infantum*	Prevalence	[51]
									Risk factors	
Gigloo et al.	2018	Iran	617	VL	IC	None	Households	*L. infantum*	Prevalence	[52]
									Risk factors	
Asfaram et al.	2017	Iran	600	VL	IC	None	Blood donors	*L. infantum*	Prevalence	[53]
Sarkari et al.	2015	Iran	2003	VL	IC	nd	Blood donors	*L. infantum*	Prevalence	[54]
Mohammadiha et al.	2013	Iran	82	VL	IC	None	Volunteers	*L. infantum*	Test evaluation	[55]
Sassi et al.	2012	Tunisia	119	VL and CL	IC	None	Volunteers	*L. infantum *and* L. major*	Test evaluation	[56]
							Households			
Saghrouni et al.	2012	Tunisia	94	VL	IC	None	Households	*L. infantum* and* L. major*	Frequency	[57]
									Leishmaniasis contacts	
Alborzi et al.	2008	Iran	388	VL	IC	None	Volunteers		Prevalence	[58]
									Test evaluation	
Fakhar et al.	2008	Iran	802	VL	IC	Yes	Households	*L. infantum*	Prevalence	[59]
Sassi et al.	1999	Tunisia	45	CL	IC		Volunteers	*L. major*	Immunological biomarkers	[60]
									Test evaluation	
Echchakery et al.	2018	Morocco	200	VL	HIV	None	Volunteers	*L. infantum*	Prevalence	[61]
Rezaei et al.	2018	Iran	251	VL	HIV	None	Volunteers	*L. infantum*	Prevalence	[62]
WHO European Region										
Molina et al.	2020	Spain	50	VL	IC	None	Blood donors	*L. infantum*	Epidemiology	[63]
Ortalli et al.	2020	Italy	240	nd	IC	None	Blood donors	*L. infantum*	Prevalence	[11]
Aliaga et al.	2019	Spain	1260	VL	IC	nd	Blood donors	*L. infantum*	Prevalence	[64]
									Risk factors	
									Epidemiology	
Ibarra-Meneses et al.	2019	Spain	805	VL	IC	None	Volunteers	*L. infantum*	Prevalence	[12]
									Risk factors	
Ibarra-Meneses et al.	2017	Spain	40	VL	IC	nd	Blood donors	*L. infantum*	Immunological biomarkers	[65]
Ibarra-Meneses et al.	2017	Spain and Bangladesh	305 and 25	VL	IC	None	Blood donors and volunteers	*L. infantum*	Immunological biomarkers	[66]
								*L. donovani*		
Ibarra-Meneses et al.	2016	Spain	47	VL	IC	nd	Blood donors	*L. infantum*	Immunological biomarkers	[67]
Pérez-Cutillas et al.	2015	Spain	657	VL	IC	nd	Blood donors	*L. infantum*	Prevalence	[68]
									Spatial distribution	
									Epidemiology	
Ates et al.	2013	Turkey	343	VL	IC	nd	Blood donors	*L. infantum*	Prevalence	[69]
									Test evaluation	
Sisko-Kraljevic et al.	2013	Croatia	2035	VL	IC	nd	Volunteers	*L. infantum*	Prevalence	[70]
Ates et al.	2012	Turkey	188	VL	IC	None	Blood donors	*L. infantum*	Prevalence	[71]
									Test evaluation	
Riera et al.	2008	Spain	1437	VL	IC	None	Blood donors	*L. infantum*	Prevalence	[72]
Scarlata et al.	2008	Italy	1449	VL	IC	None	Blood donors	*L. infantum*	Prevalence	[73]
Sakru et al.	2007	Turkey	82	VL	IC	nd	Volunteers	*L. infantum*	Prevalence	[74]
Papadopoulou et al.	2005	Greece	1200	VL	IC	None	Volunteers	*L. infantum*	Prevalence	[75]
Riera et al.	2004	Spain	656	VL	IC	nd	Blood donors	*L. infantum*	Test evaluation	[76]
Adini et al.	2003	Israel	2580	VL	IC	nd	Households	*L. donovani*	Prevalence	[77]
Fichoux et al.	1999	France	565	VL	IC	None	Blood donors	*L. infantum*	Prevalence	[78]
Federico et al.	1991	Italy	591	VL	IC	None	Blood donors	*L. infantum*	Prevalence	[79]
Botana et al.	2019	Spain	82	VL	HIV	None	Volunteers	*L. infantum*	Immunological biomarkers	[80]
Ena et al.	2014	Spain	179	VL	HIV	None	Volunteers	*L. infantum*	Prevalence	[81]
Colomba et al.	2009	Italy	145	VL	HIV	Yes	Volunteers	*L. infantum*	Prevalence	[82]
									Infection markers	
Garcia-Garcia et al.	2006	Spain	92	VL	HIV	None	Volunteers	*L. infantum*	Prevalence	[83]
									Test evaluation	
Pineda et al.	1998	Spain	291	VL	HIV	Yes	Volunteers	*L. infantum*	Prevalence	[84]
									Risk factors	
Botana et al.	2021	Spain	94	VL	IS	None	Volunteers	*L. infantum*	Immunological biomarkers	[85]
									Prevalence	
Guillen et al.	2020	Spain	192	VL	IS	None	Volunteers	*L. infantum*	Prevalence	[86]
									Disease progression	
Mary et al.	2006	France	111	VL	IC, HIV, and IS	None	Volunteers	*L. infantum*	Test evaluation	[87]
Comai et al.	2021	Italy	119	VL	SOT	None	Volunteers	*L. infantum*	Prevalence	[17]
Elmahallawy et al.	2015	Spain	625	VL	SOT	None	Volunteers	*L. infantum*	Prevalence	[88]
Region of the Americas										
Silva et al.	2020	Brazil	500	VL	IC	nd	Blood donors	*L. infantum*	Prevalence	[89]
								*L. braziliensis*		
Porcino et al.	2019	Brazil	132	VL	IC and VL	nd	Volunteers	*L. infantum*	Test evaluation	[90]
									Immunological biomarkers	
Ferreira-Silva et al.	2018	Brazil	608	VL	IC	None	Blood donors	*L. infantum*	Prevalence	[91]
Marques et al.	2017	Brazil	935	VL	IC	None	Households	*L. chagasi*	Prevalence	[92]
									Risk factors	
Medeiros et al.	2017	Brazil	33	VL	IC	None	Volunteers	*L. infantum*	Test evaluation	[93]
Braga et al.	2015	Brazil	176	CL	IC	None	Blood donors	*L. braziliensis*	Prevalence	[94]
Fukutani et al.	2014	Brazil	700	VL	IC	None	Blood donors	*L. infantum*	Prevalence	[95]
								*L. amazonensis*		
Franca et al.	2013	Brazil	430	VL	IC	None	Blood donors	*L. chagasi*	Prevalence	[96]
Silva et al.	2013	Brazil	149	VL	IC	Yes	Volunteers	*L. chagasi*	Disease progression	[97]
Añez et al.	2012	Venezuela	1036	VL	IC	None	Households	*L. infantum*	Prevalence	[98]
Santos et al.	2012	Brazil	1875	VL	IC	nd	Households	*L. infantum*	Disease progression	[99]
Lima et al.	2012	Brazil	345	VL	IC	nd	Households	*L. chagasi*	Prevalence	[100]
Carneiro et al.	2011	Brazil	1604	VL	IC	nd	Households	*L. infantum*	Test evaluation	[101]
									Disease progression	
									Epidemiology	
Silva et al.	2011	Brazil	246	VL	IC	None	Volunteers	*L. chagasi*	Disease progression	[102]
Crescente et al.	2009	Brazil	946	VL	IC	nd	Households	*L. chagasi*	Prevalence	[103]
Romero et al.	2009	Brazil	1017	VL	IC	None	Volunteers	*L. chagasi*	Test evaluation	[104]
Viana et al.	2008	Brazil	138	VL	IC	None	Volunteers	*L. chagasi*	Prevalence	[105]
									Immunological biomarkers	
									Leishmaniasis contacts	
Oliveira et al.	2008	Brazil	220	VL	IC	nd	Households	*L. chagasi*	Prevalence	[106]
									Leishmaniasis contacts	
Nascimento et al.	2006	Brazil	1016	VL	IC	Yes	Households	*L. chagasi*	Immunological biomarkers	[107]
Moreno et al.	2006	Brazil	1604	VL	IC	nd	Households	*L. chagasi*	Prevalence	[108]
									Test evaluation	
Nascimento et al.	2005	Brazil	1520	VL	IC	nd	Volunteers	*L. chagasi*	Prevalence	[109]
Braz et al.	2002	Brazil	168	VL	IC	None	Household	*L. chagasi*	Test evaluation	[110]
Caldas et al.	2001	Brazil	648	VL	IC	Yes	Households	*L. chagasi*	Prevalence	[111]
									Risk factors	
Corredor et al.	1999	Colombia	1140	VL	IC	None	Households	*L. chagasi*	Prevalence	[112]
							Indigenous		Risk factors	
									Epidemiology	
Guarin et al.	2006	Colombia	11	CL	IC	None	Volunteers	*L. panamensis*	Immunological biomarkers	[113]
								*L. amazonensis*	Test evaluation	
Torrellas et al.	2020	Venezuela	841	CL	IC	nd	Households	*L. mexicana*	Prevalence	[14]
								*L. braziliensis*		
								*L. guyanensis*		
Arraes et al.	2008	Brazil	130	CL	IC	nd	Households	*L. braziliensis*	Prevalence	[114]
									Leishmaniasis contacts	
Best et al.	2018	Peru	28	CL	IC	nd	Households	*L. braziliensis*	Immunological biomarkers	[115]
									Disease progression	
Guedes et al.	2021	Brazil	487	VL	HIV	None	Volunteers	*L. infantum*	Prevalence	[116]
Cunha et al.	2020	Brazil	240	VL	HIV	None	Volunteers	*L. infantum*	Frequency	[117]
Orsini et al.	2012	Brazil	381	VL	HIV	nd	Volunteers	*L. infantum*	Prevalence	[118]
Clemente et al.	2014	Brazil	67	VL	SOT	None	Volunteers	*L. infantum*	Prevalence	[119]

*VL* visceral leishmaniasis; *CL* cutaneous leishmaniasis; *PKDL* post-kala azar dermal leishmaniasis; *IC* immunocompetent; *HIV* human immunodeficiency virus; *SOT* solid organ transplant; *nd* not defined; *Ref.* reference

The clinical status of patients is associated with their immunological status. In this scoping review, the majority of studies (84.9%) were conducted in immunocompetent (IC) subjects. That said, 11.3% of the studies were conducted in HIV patients and 2.8% in solid organ transplant (SOT) recipients, and only 1.9% were identified as having been conducted in IS individuals. The studies in HIV patients were carried out in Spain (*n*  = 4), Brazil (*n*  = 3), Ethiopia (*n*  = 2), France (*n*  = 1), Italy (*n*  = 1), Iran (*n*  = 1), and Morocco (*n*  = 1). Meanwhile, the three studies in drug-IS populations were carried out in Spain (*n*  = 2) and France (*n*  = 1). Finally, the three studies in SOT recipients were carried out in Brazil (*n*  = 1), Italy (*n*  = 1), and Spain (*n*  = 1). Within the 84.9% of works investigating the IC population, 7.8% describe CL, and 2.2% post-kala-azar dermal leishmaniasis (PKDL). We found seven studies in IC populations with various cutaneous manifestations in seven countries: Brazil (*n*  = 2), Tunisia (*n*  = 2), Colombia (*n*  = 1), Peru (*n*  = 1), and Venezuela (*n*  = 1). Meanwhile, only two studies described PKDL (India and Bangladesh). The vast majority of studies included in this review investigated VL.

A total of 22.6% of included studies were performed in blood banks to determine the prevalence of asymptomatic infection, and 39.6% were performed in volunteer subjects where screening of the population was carried out to evaluate new tests or in clinical trials, or to find markers of exposure or progression. Finally, the remaining 37.7% of studies aimed to determine the prevalence of asymptomatic *Leishmania* infection in subjects who reported close contact with symptomatic leishmaniasis patients (households).

To include patients in blood bank studies, surveys, or clinical trials, knowledge of their previous clinical history is vital. In this scoping review, we found that 9.4% of the studies included subjects with a previous history of leishmaniasis in their asymptomatic group; 59.4% of the studies excluded those subjects who had suffered from leishmaniasis in the past, while 33% did not describe the history of leishmaniasis in the study population.

### Tests used for identification of asymptomatic *Leishmania* infection

**Fig. 4:**
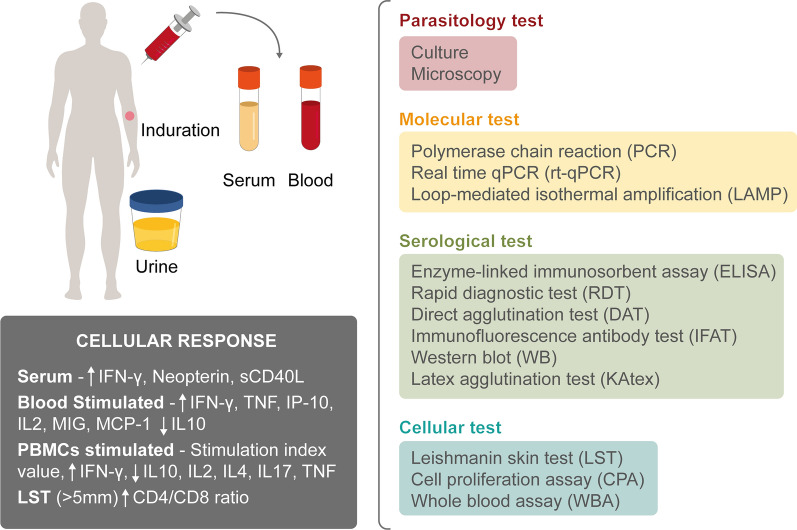
Summary of diagnostic tests employed for the detection of asymptomatic *Leishmania* infection among the included studies

The inconsistent definition of asymptomatic *Leishmania* infection further demonstrates the lack of consensus in the techniques used. Figure 4 shows an example of the different tissues (blood, serum, urine) used in combination with the tests researchers employed to define the asymptomatic population (Table 2). Parasitological (culture and microscopy), molecular (PCR, qPCR, LAMP), serological (ELISA, RDT, DAT, IFAT, WB, KAtex), and cellular (LST, CPA, WBA) techniques were employed.

Likewise, it was described that the cellular immunity of asymptomatic patients was associated with a Th1-type cellular response, where elevated levels of interferon-gamma (IFN-γ) were produced both in serum and in stimulated plasma and supernatant. It was also found that this group of asymptomatic subjects produced high levels of tumor necrosis factor (TNF), interleukin-2 (IL-2), interferon gamma-induced protein 10 (IP-10, IP-10/CXCL10), monokine induced by gamma interferon (MIG/CXCL9), monocyte chemoattractant protein-1 (MCP-1/CCL2), neopterin, and soluble CD40 ligand (sCD40L), while low levels of IL-10, IL-4, and IL-17 were detected.

### Definition of “asymptomatic infection”

As shown in Fig. 4, we report more than 10 different techniques used for the detection of the asymptomatic population in question. “Asymptomatic *Leishmania* infection” frequently describes a subject in a *Leishmania*-endemic area testing positive by a molecular or serological or cellular test, with no signs or symptoms of the disease. However, within the different groups of techniques used, there exist multiple approaches for identification and numerous test combinations for detection of asymptomatic infection. Table 3 shows the different approaches used by researchers to define asymptomatic *Leishmania* infection; 10 different ways to describe these subjects are reported. The first approach involves a combination of four techniques (serological, molecular, cellular, and parasitological). The second and third strategies involve a combination of three techniques (type 2; serological, molecular, and cellular; and type 3; serological, molecular, and parasitological). Meanwhile, types 4 (serological and molecular), 5 (serological and cellular), and 6 (molecular and cellular) used a combination of two techniques. Finally, 33 studies employed a single technique [serological (type 7), molecular (type 8), cellular (type 9), parasitological (type 10)] for the detection of the asymptomatic population in an endemic area.

**Table 3:** Strategies employed by researchers to detect asymptomatic *Leishmania* infection

Type	Test(s) used	No. of studies	References
1	Serological and molecular and cellular and parasitological	2	[72, 76]
2	Serological and molecular and cellular	13	[12, 18, 27, 51, 58, 63, 66, 80, 83, 85, 97, 100, 105]
3	Serological and molecular and parasitological	7	[35, 55, 61, 69, 78, 90, 119]
4	Serological and molecular	31	[11, 13, 17, 28, 32, 34, 41–43, 45, 46, 52–54, 59, 62, 64, 68, 73, 82, 86, 87, 91, 93, 95, 98, 99, 101, 108, 116–118]
5	Serological and cellular	14	[20, 21, 23–25, 29, 31, 49, 102, 103, 109–112]
6	Molecular and cellular	2	[14, 67]
7	Positive serological test(s) only	28	[22, 26, 30, 33, 36–39, 44, 47, 48, 50, 57, 70, 71, 74, 75, 77, 79, 81, 88, 92, 94, 96, 104, 106, 107, 114]
8	Positive molecular test(s) only	2	[41, 89]
9	Positive cellular test(s) only	5	[56, 60, 65, 113, 115]
10	Positive parasitological test only	1	[84]

### Markers for asymptomatic infection

**Fig. 5:**
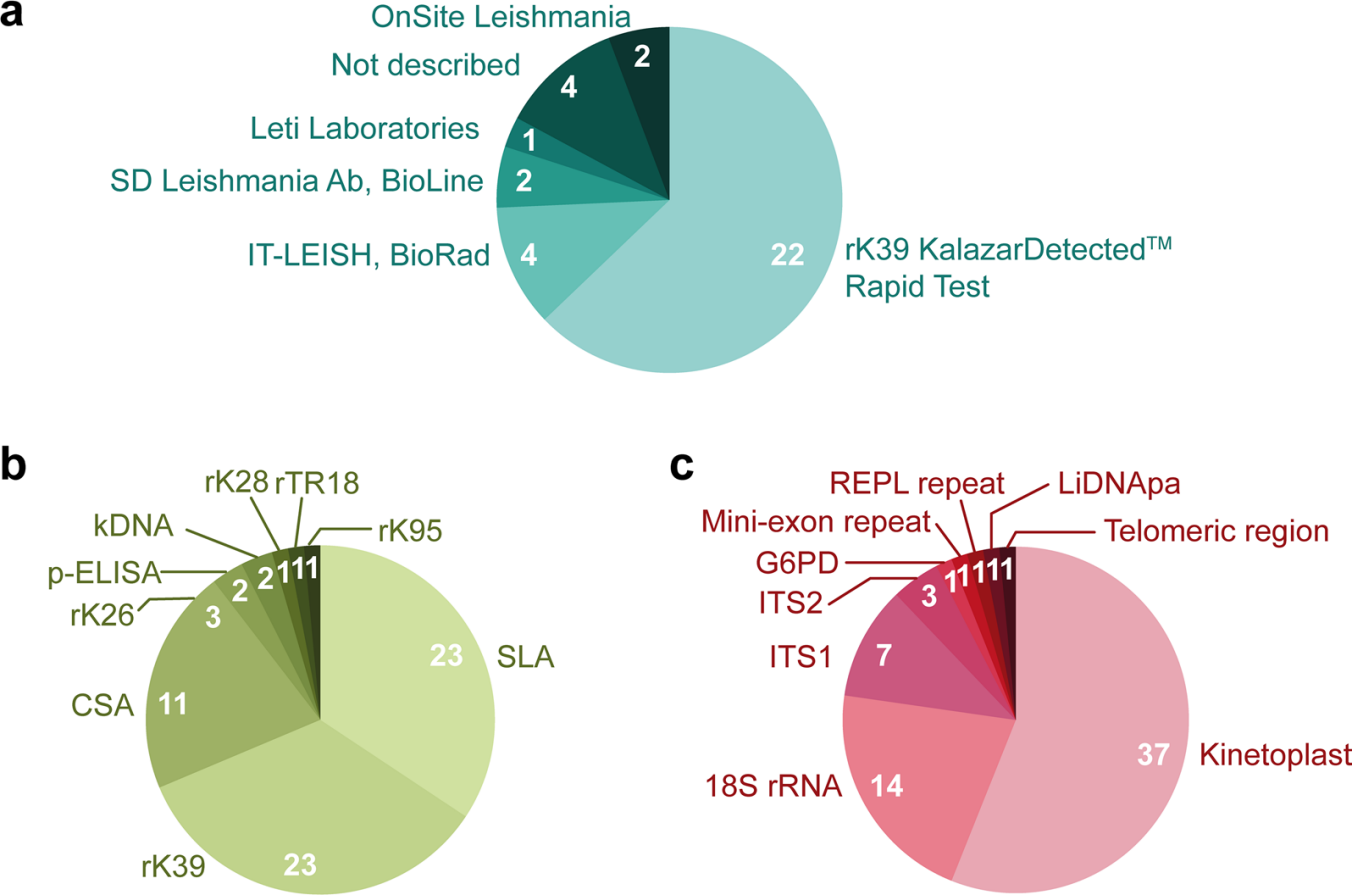
Studies included in the scoping review using **a** rapid diagnostic tests (RDT), **b** ELISA, and **c** molecular tests for detection of asymptomatic *Leishmania* infection. Pie slice size represents the percentage of tests of each type with the indicated target/brand, while numerals denote the number of papers represented by each slice

Fifty percent of the studies included in this scoping review used an ELISA for identification of asymptomatic *Leishmania* infection, followed by PCR (40%), RDT (35%), IFAT (30%), DAT (29%), and LST (22%). To determine how multiple tests are applied in parallel, we identified 78 eligible studies where at least two tests were used on either the entire population or a subset thereof. Inspection of the network (Fig. 5) showed a high degree of interconnectivity between several of the tests. The highest density of interconnectivity was observed for DAT, RDT, IFAT, PCR, and ELISA, where links were found to all other tests. LAMP was the test method with the least interconnectivity within the network, linking only with ELISA, RDT, IFAT, and PCR once each.

**Fig. 6:**
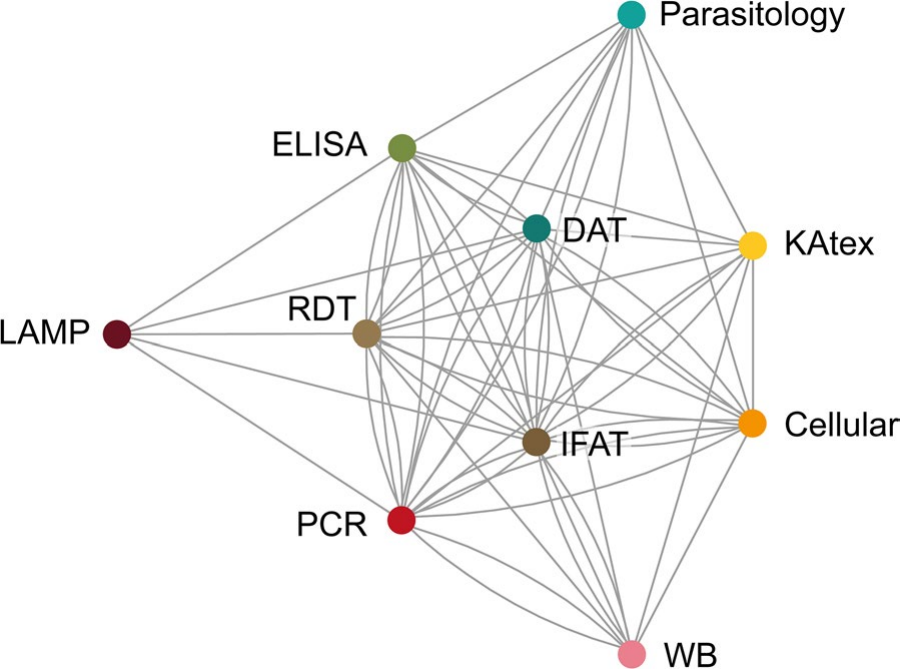
Network demonstrating comparisons of various tests employed for the detection of asymptomatic *Leishmania* infection within articles included in the scoping review. In the network diagram, the nodes represent the test type and the edges (lines) indicate that two test types have been compared in at least one study

A combination of serological and molecular techniques was the most common combination of detection approaches among the studies included in this scoping review. However, it is important to note that the results mentioned above do not consider the antigen or target used. Figure 6a summarizes the diversity of commercial brands that exist for the rK39 rapid diagnostic test (rK39-RDT). Sixty-three percent of the studies that employed RDT used the rK39 Kalazar *Detect*™ rapid test (InBios), while 11% used IT Leish (BioRad), 6% used the SD BIOLINE *Leishmania* Ab Test (Bioline/Abbott), 6% used OnSite *Leishmania* IgM/IgG Combo test (CTK Biotech, Inc.), 3% used the Leti Laboratories test, and 11% of articles did not describe the commercial brand in question. Figure 6b shows the diversity of antigens employed for the detection of antibodies by ELISA. Thirty-four percent of the studies that used ELISA for identification of the asymptomatic population used soluble *Leishmania* antigen (SLA), another 34% used the rK39 antigen, while 16% used crude *Leishmania* antigen (CSA), 4% used recombinant kinesin 26 (rK26) antigen, 3% used *Leishmania* promastigotes, and 1% used recombinant kinesin 28 (rK28), rTR18, and rKR95 antigen. For the molecular techniques described (Fig. 6c), nine different targets were identified. Of these, 57% of studies used kinetoplast DNA as their target (kDNA), 21% the small subunit *18S rRNA* gene (*ssu* 18S rRNA), and 11% and 3% used internal transcribed spacer (ITS) 1 and 2, respectively. Meanwhile, 1% of studies employed the glucose-6-phosphate dehydrogenase gene (*g6pd* gene), mini-exon repeat, REPL repeat, DNA polymerase alpha (DNApα), and the telomeric region.

## Discussion

Our scoping review included a total of 106 articles from 19 countries in five of six different WHO regions. There has been a marked increase in studies conducted on the subject of asymptomatic *Leishmania* infections in recent years (2017–2021) versus past decades, possibly due to increased awareness of their potential significance in the epidemiology of leishmaniasis. Most of the studies included in our review were conducted in Brazil (26%) and India (12%); this is likely because leishmaniasis is very widespread in these countries, which constitute two of the six countries responsible for more than 90% of VL cases around the world [3]. The vast majority (84.9%) of studies included in this review explore asymptomatic *Leishmania* infection in IC populations. Different mathematical modeling studies have shown that this asymptomatic IC population is less infective than the population with active disease, and their role in disease transmission is still under investigation [15, 63]. The issue remains of major concern and could have a substantial impact on the spread of this parasite [63, 91].

Notably, leishmaniasis is of much greater risk to IS populations, including HIV-positive individuals, SOT recipients, and patients under treatment with immunosuppressive drugs. HIV/*Leishmania* co-infections more often lead to clinical VL, and with greater severity [117, 120]. Indeed, it has previously been demonstrated that HIV-infected individuals with asymptomatic *Leishmania* infection may transmit the parasite to sand flies, leading to further spread of infection [63, 121]. That said, in many studies, the number of participants is too low to confirm results. Of note, the majority of the studies performed in an HIV-positive population included in this scoping review were conducted in the WHO European Region, the area where most HIV cases have been reported [120]. However, over the years, there has been an increase in HIV cases in other *Leishmania*-endemic areas, such as Brazil, East Africa, and India [13, 116].

Given the likely significance of the asymptomatic IS population in *Leishmania* epidemiology as described above, knowing the prevalence of HIV infection (and furthermore, exploring biomarkers for VL progression) in *Leishmania*-endemic areas becomes ever more important; interestingly, the studies included in our review used up to 13 tests and six different approaches to define infection in this population (see Table 3). The large number of tests and strategies employed underscores the difficulty in defining asymptomatic infection in HIV patients in endemic areas. Interestingly, one research group used only a single parasitological test for this purpose. Pineda et al. describe an asymptomatic HIV patient as one in whom amastigotes are detected in bone marrow aspirate samples [84]. This is in fact the gold standard for diagnosis of *Leishmania*; however, bone marrow aspirate is an invasive approach [122]. Currently, in the WHO road map, minimally invasive techniques that enable detection of this population (and therefore safe and efficient establishment of new control measures) are prioritized [123].

With respect to populations under treatment with immunosuppressive drugs, despite the low number of studies reported (*n*  = 3), seven different tests and two different strategies were employed for detection of the asymptomatic population [85–87]. This same pattern was found with SOT recipients: five different tests and three different strategies were used in the three studies reported in this scoping review [17, 88, 119]. These data confirm the lack of consensus on defining and detecting asymptomatic infection in these populations.

In total, this scoping review identified 14 different tests used for detection of parasites, parasite load, antibodies/antigens, and cellular immune response, resulting in nine different overall approaches for defining asymptomatic infections in IC populations. There is a complex relationship between the various tests used for the detection of asymptomatic populations, as demonstrated by the interconnectivity in our network. The nine strategies employed can be separated into two main categories: strategies 1–6 involved independent combinations of serological, molecular, cellular, and/or parasitological tests to identify the asymptomatic cohort; on the other hand, strategies 7, 8, and 9 involved the use of a single test type to detect asymptomatic individuals. These two very different, wide categories broaden the detection range, leading to a lack of consensus concerning the identification of the asymptomatic population in *Leishmania*-endemic areas. Furthermore, the use of different strategies makes it difficult to compare the same population in the same (or different) endemic areas. Importantly, this complication lies not only in the large number of tests employed, but also in the numerous targets (molecular tests) or antigens (serological and cellular tests) used by each group. We report the use of more than six types of rapid test (rK39-RDT) from different manufacturers, and the effectiveness of these commercial tests varies between regions [124]. Moreover, nine different antigens were utilized to detect antibodies using ELISA alone [125], and nine different targets were employed to identify the parasite with molecular tools [126]. This substantial variation in detection methods coupled with the plethora of definitions of “asymptomatic *Leishmania* infection” makes an accurate determination of prevalence in any region near impossible.

### Common usage guidelines

Standardization of the term “asymptomatic *Leishmania* infection” is key to improving the outcome of future studies and allowing accurate comparison between the results of different works. According to the literature, subjects are often considered as asymptomatically infected with *Leishmania* under the following conditions: residence (or extended stay) in a *Leishmania*-endemic area, no reported signs/symptoms compatible with leishmaniasis, and positive on a combination of serological, molecular, cellular, and/or parasitological tests. We consider this an appropriate definition. While a medical history of leishmaniasis may complicate interpretation of certain tests, we feel it does not preclude the development of an asymptomatic infection if the subject was previously considered “cured.” More research regarding how many years following a reported VL episode a patient can be considered “asymptomatic” is necessary; as such, it is recommended that the conditions of a study population with VL history be explicitly stated. Furthermore, we recommend caution when comparing results of different studies on the subject of asymptomatic infections, as the reported prevalence cannot confidently be compared between areas due to the wide variety of tests employed by research groups. With respect to future studies, we suggest that researchers use the most sensitive and specific test available to them, and interpret their results within the framework of other studies employing the same technique and target. We recommend a minimum of two different detection methods employed in parallel for identification of asymptomatic *Leishmania* infections among a study population.

Of note, for this work, we did not analyze the results/methodology of each piece of literature included in the review; this was beyond the scope of our study. There may therefore be limitations with respect to the quality of the methodology employed in each individual article. In the future, we will perform a meta-analysis in order to compare the sensitivity and specificity of different diagnostic tests employed by research groups for the detection of asymptomatic *Leishmania*-infected populations in endemic areas (Table 4).

**Table 4:** Gaps and opportunities associated with asymptomatic *Leishmania* infection

Gaps
Lack of consensus regarding definition of asymptomatic infection
Lack of consensus regarding optimal technique for identification of asymptomatic population
Large variety of test targets and antigens employed by different research groups
Lack of knowledge pertaining to the potential role of asymptomatic individuals in *Leishmania* disease transmission and epidemiology
Lack of knowledge pertaining to the factors associated with development of clinical leishmaniasis by individuals previously considered to be asymptomatically infected
Opportunities
Establish a standard definition of “asymptomatic *Leishmania* infection”
Determine the optimal technique for identification of the asymptomatic population (technique, target/antigen)
Determine the true prevalence of asymptomatic *Leishmania* infection in different regions
Determine the true role of asymptomatic *Leishmania*-infected subjects (both immunocompetent and immunosuppressed) in transmission of leishmaniasis
Establish objective, quantifiable markers associated with the development of clinical leishmaniasis by previously asymptomatically infected individuals (differentiate subclinical and asymptomatic infections)
Determine the principal risk factors related to development of clinical leishmaniasis

## Conclusions

Asymptomatic *Leishmania* infection remains poorly understood; the lack of baseline tests for its detection means that its prevalence is likely underestimated, and its epidemiological role remains unknown. This scoping review was performed in order to inform researchers of the different approaches that exist for identification of asymptomatic *Leishmania* infection. It also highlights the need to standardize the definition of this population in order to reach a consensus for future work strategies in endemic areas, especially in IS populations.

## Supplementary Information

**Additional file 1: Table S1.** List of countries considered endemic.

**Additional file 2: Table S2.** Search syntax for PubMed.

## Abbreviations

CL: Cutaneous leishmaniasis
DAT: Direct agglutination test
CPA: Cell proliferation assay
CSA: Crude *Leishmania* antigen
DNApα: DNA polymerase alpha
ELISA: Enzyme-linked immunosorbent assay
*g6pd*: Glucose-6-phosphate dehydrogenase
HIV: Human immunodeficiency virus
IGRA: Interferon-gamma release assay
IC: Immunocompetent
IFAT: Immunofluorescence antibody test
IFN-γ: Interferon-gamma
IL-2: Interleukin-2
IP-10: Interferon gamma-induced protein 10
ITS: Internal transcribed spacer
IS: Immunosuppressed
kDNA: Kinetoplast DNA
LAMP: Loop-mediated isothermal amplification
LST: Leishmanin skin test
MeSH: Medical Subject Headings
MIG: Monokine induced by gamma interferon
NTD: Neglected tropical disease
PCR: Polymerase chain reaction
PKDL: Post-kala-azar dermal leishmaniasis
PRISMA-P: Preferred Reporting Items for Systematic Review and Meta-Analysis Protocols
qPCR: Quantitative polymerase chain reaction
RDT: Rapid diagnostic test
rK26: Recombinant kinesin 26
rK28: Recombinant kinesin 28
rK39-RDT: Recombinant kinesin 39 rapid diagnostic test
SOT: Solid organ transplant
sCD40L: SolubleCD40 ligand
*ssu*18S rRNA: Small subunit 18S ribosomal RNA
TNF: Tumor necrosis factor
VL: Visceral leishmaniasis
WB: Western blot
WBA: Whole blood assay
WHO: World Health Organization

## References

[1] WHO (2010). Control of the leishmaniases. World Health Organization technical report series.

[2] Ashford RW (1996). Leishmaniasis reservoirs and their significance in control. Clin Dermatol.

[3] Alvar J, Velez ID, Bern C, Herrero M, Desjeux P, Cano J (2012). Leishmaniasis worldwide and global estimates of its incidence. PLoS ONE.

[4] Burza S, Croft SL, Boelaert M (2018). Leishmaniasis. Lancet.

[5] Singh OP, Hasker E, Boelaert M, Sacks D, Sundar S (2020). Xenodiagnosis to address key questions in visceral leishmaniasis control and elimination. PLoS Negl Trop Dis.

[6] Badaro R, Jones TC, Carvalho EM, Sampaio D, Reed SG, Barral A (1986). New perspectives on a subclinical form of visceral leishmaniasis. J Infect Dis.

[7] Michel G, Pomares C, Ferrua B, Marty P (2011). Importance of worldwide asymptomatic carriers of *Leishmania**infantum* (*L.**chagasi*) in human. Acta Trop.

[8] Singh OP, Hasker E, Sacks D, Boelaert M, Sundar S (2014). Asymptomatic *Leishmania* infection: a new challenge for *Leishmania* control. Clin Infect Dis.

[9] Hasker E, Kansal S, Malaviya P, Gidwani K, Picado A, Singh RP (2013). Latent infection with *Leishmania donovani* in highly endemic villages in Bihar. India PLoS Negl Trop Dis.

[10] Owen SI, Hossain F, Ghosh P, Chowdhury R, Hossain MS, Jewell C (2021). Detection of asymptomatic *Leishmania* infection in Bangladesh by antibody and antigen diagnostic tools shows an association with post-kala-azar dermal leishmaniasis (PKDL) patients. Parasit Vectors.

[11] Ortalli M, De Pascali AM, Longo S, Pascarelli N, Porcellini A, Ruggeri D (2020). Asymptomatic *Leishmania infantum* infection in blood donors living in an endemic area, northeastern Italy. J Infect.

[12] Ibarra-Meneses AV, Carrillo E, Nieto J, Sanchez C, Ortega S, Estirado A (2019). Prevalence of asymptomatic *Leishmania* infection and associated risk factors, after an outbreak in the south-western Madrid region, Spain, 2015. Euro Surveill.

[13] van Griensven J, van Henten S, Mengesha B, Kassa M, Adem E, Endris Seid M (2019). Longitudinal evaluation of asymptomatic *Leishmania* infection in HIV-infected individuals in North-West Ethiopia: a pilot study. PLoS Negl Trop Dis.

[14] Torrellas A, Ferrer E, Cruz I, De Lima H, Borges R, Delgado O (2020). Surveillance for *Leishmania* asymptomatic infection in endemic foci of cutaneous leishmaniasis in Venezuela: a combination of leishmanin skin test and PCR using blood clots improves detection and enables identification of species. Trans R Soc Trop Med Hyg.

[15] Stauch A, Sarkar RR, Picado A, Ostyn B, Sundar S, Rijal S (2011). Visceral leishmaniasis in the Indian subcontinent: modelling epidemiology and control. PLoS Negl Trop Dis.

[16] StataCorp (2013). Stata statistical software: release 13.

[17] Comai G, De Pascali MA, Busutti M, Morini S, Ortalli M, Conte D (2021). Screening strategies for the diagnosis of asymptomatic *Leishmania* infection in dialysis patients as a model for kidney transplant candidates. J Nephrol.

[18] Chakravarty J, Hasker E, Kansal S, Singh OP, Malaviya P, Singh AK (2019). Determinants for progression from asymptomatic infection to symptomatic visceral leishmaniasis: a cohort study. PLoS Negl Trop Dis.

[19] Tricco AC, Lillie E, Zarin W, O'Brien KK, Colquhoun H, Levac D (2018). PRISMA extension for scoping reviews (PRISMA-ScR): checklist and explanation. Ann Intern Med.

[20] Bejano S, Shumie G, Kumar A, Asemahagn E, Damte D, Woldie S (2021). Prevalence of asymptomatic visceral leishmaniasis in human and dog, Benishangul Gumuz regional state, Western Ethiopia. Parasit Vectors.

[21] Tadese D, Hailu A, Bekele F, Belay S (2019). An epidemiological study of visceral leishmaniasis in North East Ethiopia using serological and leishmanin skin tests. PLoS ONE.

[22] Ayehu A, Aschale Y, Lemma W, Alebel A, Worku L, Jejaw A (2018). Seroprevalence of asymptomatic *Leishmania donovani* among laborers and associated risk factors in agricultural camps of West Armachiho District, Northwest Ethiopia: a cross-sectional study. J Parasitol Res.

[23] Custodio E, Gadisa E, Sordo L, Cruz I, Moreno J, Nieto J (2012). Factors associated with *Leishmania* asymptomatic infection: results from a cross-sectional survey in highland northern Ethiopia. PLoS Negl Trop Dis.

[24] Gadisa E, Custodio E, Canavate C, Sordo L, Abebe Z, Nieto J (2012). Usefulness of the rK39-immunochromatographic test, direct agglutination test, and leishmanin skin test for detecting asymptomatic *Leishmania* infection in children in a new visceral leishmaniasis focus in Amhara State, Ethiopia. Am J Trop Med Hyg.

[25] Adriaensen W, Abdellati S, van Henten S, Gedamu Y, Diro E, Vogt F (2018). Serum levels of soluble CD40 ligand and neopterin in HIV coinfected asymptomatic and symptomatic visceral leishmaniasis patients. Front Cell Infect Microbiol.

[26] Basnyat S, Banjara MR, Ghimire P, Matlashewski G, Singh A (2021). Seropositivity of visceral leishmaniasis on people of VL endemic three districts of Nepal. Parasitol Int.

[27] Cloots K, Singh OP, Singh AK, Van der Auwera G, Kumar P, Gedda MR (2021). Assessing *L.**donovani* skin parasite load: a proof of concept study of a microbiopsy device in an Indian setting. Front Cell Infect Microbiol.

[28] Owen SI, Burza S, Kumar S, Verma N, Mahajan R, Harshana A (2021). Evaluation of qPCR on blood and skin microbiopsies, peripheral blood buffy coat smear, and urine antigen ELISA for diagnosis and test of cure for visceral leishmaniasis in HIV-coinfected patients in India: a prospective cohort study. BMJ Open.

[29] Johanson GH, Amato VS, Ribeiro VST, Tuon FF (2020). Estimation of *Leishmania* spp. infection in asymptomatic people from Muzaffarpur, Bihar, India by antigen-antibody and skin testing. Rev Inst Med Trop Sao Paulo.

[30] Mondal D, Ghosh P, Chowdhury R, Halleux C, Ruiz-Postigo JA, Alim A (2019). Relationship of serum antileishmanial antibody with development of visceral leishmaniasis, post-kala-azar dermal leishmaniasis and visceral leishmaniasis relapse. Front Microbiol.

[31] Singh AK, Das VNR, Amit A, Dikhit MR, Mahantesh V, Kumar A (2018). Identification of clinical immunological determinants in asymptomatic VL and post kala-azar dermal leishmaniasis patients. Iran J Parasitol.

[32] Kaushal H, Bhattacharya SK, Verma S, Salotra P (2017). Serological and molecular analysis of *Leishmania* infection in healthy individuals from two Districts of West Bengal, India, endemic for visceral leishmaniasis. Am J Trop Med Hyg.

[33] Saha P, Ganguly S, Chatterjee M, Das SB, Kundu PK, Guha SK (2017). Asymptomatic leishmaniasis in kala-azar endemic areas of Malda district, West Bengal. India PLoS Negl Trop Dis.

[34] Banu SS, Ahmed BN, Shamsuzzaman AKM, Lee R (2016). Evaluation of recombinant K39 antigen and various promastigote antigens in sero-diagnosis of visceral leishmaniasis in Bangladesh. Parasite Epidemiol Control.

[35] Banu SS, Meyer W, Ahmed BN, Kim R, Lee R (2016). Detection of *Leishmania donovani* in peripheral blood of asymptomatic individuals in contact with patients with visceral leishmaniasis. Trans R Soc Trop Med Hyg.

[36] Das VN, Pandey RN, Siddiqui NA, Chapman LA, Kumar V, Pandey K (2016). Longitudinal study of transmission in households with visceral leishmaniasis, asymptomatic infections and PKDL in highly endemic villages in Bihar. India PLoS Negl Trop Dis.

[37] Timilsina S, Raj Bhattarai N, Khanal B, Rijal S (2016). Serological assessment for *Leishmania donovani* infection in blood donors of Sunsari District, Dharan, Nepal. Indian J Hematol Blood Transfus.

[38] Vallur AC, Reinhart C, Mohamath R, Goto Y, Ghosh P, Mondal D (2016). Accurate serodetection of asymptomatic *Leishmania donovani* infection by use of defined antigens. J Clin Microbiol.

[39] Picado A, Ostyn B, Singh SP, Uranw S, Hasker E, Rijal S (2014). Risk factors for visceral leishmaniasis and asymptomatic *Leishmania donovani* infection in India and Nepal. PLoS ONE.

[40] Sudarshan M, Sundar S (2014). Parasite load estimation by qPCR differentiates between asymptomatic and symptomatic infection in Indian visceral leishmaniasis. Diagn Microbiol Infect Dis.

[41] Sudarshan M, Singh T, Singh AK, Chourasia A, Singh B, Wilson ME (2014). Quantitative PCR in epidemiology for early detection of visceral leishmaniasis cases in India. PLoS Negl Trop Dis.

[42] Huda MM, Rudra S, Ghosh D, Bhaskar KR, Chowdhury R, Dash AP (2013). Low prevalence of *Leishmania donovani* infection among the blood donors in kala-azar endemic areas of Bangladesh. BMC Infect Dis.

[43] Srivastava P, Gidwani K, Picado A, Van der Auwera G, Tiwary P, Ostyn B (2013). Molecular and serological markers of *Leishmania donovani* infection in healthy individuals from endemic areas of Bihar. India Trop Med Int Health.

[44] Ostyn B, Gidwani K, Khanal B, Picado A, Chappuis F, Singh SP (2011). Incidence of symptomatic and asymptomatic *Leishmania donovani* infections in high-endemic foci in India and Nepal: a prospective study. PLoS Negl Trop Dis.

[45] Topno RK, Das VN, Ranjan A, Pandey K, Singh D, Kumar N (2010). Asymptomatic infection with visceral leishmaniasis in a disease-endemic area in Bihar. India Am J Trop Med Hyg.

[46] Bhattarai NR, Van der Auwera G, Khanal B, De Doncker S, Rijal S, Das ML (2009). PCR and direct agglutination as *Leishmania* infection markers among healthy Nepalese subjects living in areas endemic for kala-azar. Trop Med Int Health.

[47] Gidwani K, Kumar R, Rai M, Sundar S (2009). Longitudinal seroepidemiologic study of visceral leishmaniasis in hyperendemic regions of Bihar. India Am J Trop Med Hyg.

[48] Sinha PK, Bimal S, Pandey K, Singh SK, Ranjan A, Kumar N (2008). A community-based, comparative evaluation of direct agglutination and rK39 strip tests in the early detection of subclinical *Leishmania donovani* infection. Ann Trop Med Parasitol.

[49] Bern C, Haque R, Chowdhury R, Ali M, Kurkjian KM, Vaz L (2007). The epidemiology of visceral leishmaniasis and asymptomatic leishmanial infection in a highly endemic Bangladeshi village. Am J Trop Med Hyg.

[50] Chowdhury MS, el Harith A, al Massum A, al Karim E, al Rahman A (1993). Prevalence of agglutinating anti-*Leishmania* antibodies in two multi-thousand Bengoli communities. Parasitol Res.

[51] Mody RM, Lakhal-Naouar I, Sherwood JE, Koles NL, Shaw D, Bigley DP (2019). Asymptomatic visceral *Leishmania infantum* infection in US soldiers deployed to Iraq. Clin Infect Dis.

[52] Layegh Gigloo A, Sarkari B, Rezaei Z, Hatam GR, Davami MH (2018). Asymptomatic *Leishmania* infected children: a seroprevalence and molecular survey in a rural area of Fars Province, Southern Iran. J Trop Med.

[53] Asfaram S, Fakhar M, Mohebali M, Mardani A, Banimostafavi ES, Ziaei Hezarjaribi H (2017). Asymptomatic human blood donors carriers of *Leishmania infantum*: potential reservoirs for visceral leishmaniasis in northwestern Iran. Transfus Apher Sci.

[54] Sarkari B, Gadami F, Shafiei R, Motazedian MH, Sedaghat F, Kasraian L (2015). Seroprevalence of *Leishmania* infection among the healthy blood donors in kala-azar endemic areas of Iran. J Parasit Dis.

[55] Mohammadiha A, Mohebali M, Haghighi A, Mahdian R, Abadi AR, Zarei Z (2013). Comparison of real-time PCR and conventional PCR with two DNA targets for detection of *Leishmania* (*Leishmania*) *infantum* infection in human and dog blood samples. Exp Parasitol.

[56] Sassi A, Ben Salah A, Hamida NB, Zaatour A (2012). Age related efficiency of the leishmanin skin test as a marker of immunity to human visceral leishmaniasis. Arch Inst Pasteur Tunis.

[57] Saghrouni F, Khammari I, Kaabia N, Bouguila J, Ben Abdeljelil J, Fathallah A (2012). Asymptomatic carriage of *Leishmania* in family members of patients with visceral leishmaniasis in Central Tunisia. Pathol Biol.

[58] Alborzi A, Pourabbas B, Shahian F, Mardaneh J, Pouladfar GR, Ziyaeyan M (2008). Detection of *Leishmania infantum* kinetoplast DNA in the whole blood of asymptomatic individuals by PCR-ELISA and comparison with other infection markers in endemic areas, southern Iran. Am J Trop Med Hyg.

[59] Fakhar M, Motazedian MH, Hatam GR, Asgari Q, Kalantari M, Mohebali M (2008). Asymptomatic human carriers of *Leishmania infantum*: possible reservoirs for Mediterranean visceral leishmaniasis in southern Iran. Ann Trop Med Parasitol.

[60] Sassi A, Louzir H, Ben Salah A, Mokni M, Ben Osman A, Dellagi K (1999). Leishmanin skin test lymphoproliferative responses and cytokine production after symptomatic or asymptomatic *Leishmania major* infection in Tunisia. Clin Exp Immunol.

[61] Echchakery M, Nieto J, Boussaa S, El Fajali N, Ortega S, Souhail K (2018). Asymptomatic carriers of *Leishmania infantum* in patients infected with human immunodeficiency virus (HIV) in Morocco. Parasitol Res.

[62] Rezaei Z, Sarkari B, Dehghani M, Layegh Gigloo A, Afrashteh M (2018). High frequency of subclinical *Leishmania* infection among HIV-infected patients living in the endemic areas of visceral leishmaniasis in Fars province, southern Iran. Parasitol Res.

[63] Molina R, Jimenez M, Garcia-Martinez J, San Martin JV, Carrillo E, Sanchez C (2020). Role of asymptomatic and symptomatic humans as reservoirs of visceral leishmaniasis in a Mediterranean context. PLoS Negl Trop Dis.

[64] Aliaga L, Ceballos J, Sampedro A, Cobo F, Lopez-Nevot MA, Merino-Espinosa G (2019). Asymptomatic *Leishmania* infection in blood donors from the Southern of Spain. Infection.

[65] Ibarra-Meneses AV, Sanchez C, Alvar J, Moreno J, Carrillo E (2017). Monocyte chemotactic protein 1 in plasma from soluble *Leishmania* antigen-stimulated whole blood as a potential biomarker of the cellular immune response to *Leishmania infantum*. Front Immunol.

[66] Ibarra-Meneses AV, Ghosh P, Hossain F, Chowdhury R, Mondal D, Alvar J (2017). IFN-gamma, IL-2, IP-10, and MIG as biomarkers of exposure to *Leishmania* spp., and of cure in human visceral leishmaniasis. Front Cell Infect Microbiol.

[67] Ibarra-Meneses AV, Carrillo E, Sanchez C, Garcia-Martinez J, Lopez Lacomba D, San Martin JV (2016). Interleukin-2 as a marker for detecting asymptomatic individuals in areas where *Leishmania infantum* is endemic. Clin Microbiol Infect.

[68] Perez-Cutillas P, Goyena E, Chitimia L, De la Rua P, Bernal LJ, Fisa R (2015). Spatial distribution of human asymptomatic *Leishmania infantum* infection in southeast Spain: a study of environmental, demographic and social risk factors. Acta Trop.

[69] Ates SC, Bagirova M, Allahverdiyev AM, Kocazeybek B, Kosan E (2013). Utility of the microculture method for *Leishmania* detection in non-invasive samples obtained from a blood bank. Acta Trop.

[70] Sisko-Kraljevic K, Jeroncic A, Mohar B, Punda-Polic V (2013). Asymptomatic *Leishmania infantum* infections in humans living in endemic and non-endemic areas of Croatia, 2007 to 2009. Euro Surveill.

[71] Ates SC, Bagirova M, Allahverdiyev AM, Baydar SY, Koc RC, Elcicek S (2012). Detection of antileishmanial antibodies in blood sampled from blood bank donors in Istanbul. Future Microbiol.

[72] Riera C, Fisa R, Lopez-Chejade P, Serra T, Girona E, Jimenez M (2008). Asymptomatic infection by *Leishmania infantum* in blood donors from the Balearic Islands (Spain). Transfusion.

[73] Scarlata F, Vitale F, Saporito L, Reale S, Vecchi VL, Giordano S (2008). Asymptomatic *Leishmania infantum*/*chagasi* infection in blood donors of western Sicily. Trans R Soc Trop Med Hyg.

[74] Sakru N, Korkmaz M, Ozbel Y, Ertabaklar H, Sengul M, Toz SO (2007). Investigation of asymptomatic visceral leishmaniasis cases using western blot in an endemic area in Turkey. New Microbiol.

[75] Papadopoulou C, Kostoula A, Dimitriou D, Panagiou A, Bobojianni C, Antoniades G (2005). Human and canine leishmaniasis in asymptomatic and symptomatic population in Northwestern Greece. J Infect.

[76] Riera C, Fisa R, Udina M, Gallego M, Portus M (2004). Detection of *Leishmania infantum* cryptic infection in asymptomatic blood donors living in an endemic area (Eivissa, Balearic Islands, Spain) by different diagnostic methods. Trans R Soc Trop Med Hyg.

[77] Adini I, Ephros M, Chen J, Jaffe CL (2003). Asymptomatic visceral leishmaniasis, northern Israel. Emerg Infect Dis.

[78] le Fichoux Y, Quaranta JF, Aufeuvre JP, Lelievre A, Marty P, Suffia I (1999). Occurrence of *Leishmania infantum* parasitemia in asymptomatic blood donors living in an area of endemicity in southern France. J Clin Microbiol.

[79] Federico G, Damiano F, Caldarola G, Fantini C, Fiocchi V, Ortona L (1991). A seroepidemiological survey on *Leishmania infantum* infection. Eur J Epidemiol.

[80] Botana L, Ibarra-Meneses AV, Sanchez C, Castro A, San Martin JV, Molina L (2019). Asymptomatic immune responders to *Leishmania* among HIV positive patients. PLoS Negl Trop Dis.

[81] Ena J, Pasquau F, del Mar L-P, Martinez-Peinado C, Arjona F (2014). Screening for subclinical *Leishmania* infection in HIV-infected patients living in eastern Spain. Pathog Glob Health.

[82] Colomba C, Saporito L, Vitale F, Reale S, Vitale G, Casuccio A (2009). Cryptic *Leishmania infantum* infection in Italian HIV infected patients. BMC Infect Dis.

[83] Garcia-Garcia JA, Martin-Sanchez J, Gallego M, Rivero-Roman A, Camacho A, Riera C (2006). Use of noninvasive markers to detect *Leishmania* infection in asymptomatic human immunodeficiency virus-infected patients. J Clin Microbiol.

[84] Pineda JA, Gallardo JA, Macias J, Delgado J, Regordan C, Morillas F (1998). Prevalence of and factors associated with visceral leishmaniasis in human immunodeficiency virus type 1-infected patients in southern Spain. J Clin Microbiol.

[85] Botana L, Ibarra-Meneses AV, Sanchez C, Matia B, San Martin JV, Moreno J (2021). Leishmaniasis: a new method for confirming cure and detecting asymptomatic infection in patients receiving immunosuppressive treatment for autoimmune disease. PLoS Negl Trop Dis.

[86] Guillen MC, Alcover MM, Borruel N, Sulleiro E, Salvador F, Berenguer D (2020). *Leishmania infantum* asymptomatic infection in inflammatory bowel disease patients under anti-TNF therapy. Heliyon.

[87] Mary C, Faraut F, Drogoul MP, Xeridat B, Schleinitz N, Cuisenier B (2006). Reference values for *Leishmania infantum* parasitemia in different clinical presentations: quantitative polymerase chain reaction for therapeutic monitoring and patient follow-up. Am J Trop Med Hyg.

[88] Elmahallawy EK, Cuadros-Moronta E, Liebana-Martos MC, Rodriguez-Granger JM, Sampedro-Martinez A, Agil A (2015). Seroprevalence of *Leishmania* infection among asymptomatic renal transplant recipients from southern Spain. Transpl Infect Dis.

[89] Silva LP, Montenegro S, Werkauser R, Sales K, Soares FCS, Costa VMA (2020). Asymptomatic *Leishmania* infection in blood donors from a major blood bank in Northeastern Brazil: a cross-sectional study. Rev Inst Med Trop Sao Paulo.

[90] Porcino GN, Carvalho KSS, Braz DC, Costa Silva V, Costa CHN, de Miranda Santos IKF (2019). Evaluation of methods for detection of asymptomatic individuals infected with *Leishmania infantum* in the state of Piaui, Brazil. PLoS Negl Trop Dis.

[91] Ferreira-Silva MM, Teixeira LAS, Tiburcio MS, Pereira GA, Rodrigues V, Palis M (2018). Socio-epidemiological characterisation of blood donors with asymptomatic *Leishmania infantum* infection from three Brazilian endemic regions and analysis of the transfusional transmission risk of visceral leishmaniasis. Transfus Med.

[92] Da Santos Marques LH, Da Rocha IC, Reis IA, da Cunha GMR, Oliveira E, Pfeilsticker TR (2017). *Leishmania infantum*: illness, transmission profile and risk factors for asymptomatic infection in an endemic metropolis in Brazil. Parasitology.

[93] Medeiros FA, Gomes LI, Oliveira E, de Souza CS, Mourao MV, Cota GF (2017). Development and validation of a PCR-ELISA for the diagnosis of symptomatic and asymptomatic infection by *Leishmania* (*Leishmania*) *infantum*. J Trop Med.

[94] Braga Lde S, Navasconi TR, Leatte EP, Skraba CM, Silveira TG, Ribas-Silva RC (2015). Presence of anti-*Leishmania* (*Viannia*) *braziliensis* antibodies in blood donors in the West-Central region of the State of Parana, Brazil. Rev Soc Bras Med Trop.

[95] Fukutani KF, Figueiredo V, Celes FS, Cristal JR, Barral A, Barral-Netto M (2014). Serological survey of *Leishmania* infection in blood donors in Salvador, Northeastern Brazil. BMC Infect Dis.

[96] Franca ADDC, Landgraf V, Lima MS, Pontes E, Dorval ME (2013). Anti-*Leishmania* antibodies in blood donors from the Midwest region of Brazil. Transfus Apher Sci.

[97] Silva LA, Romero HD, Fagundes A, Nehme N, Fernandes O, Rodrigues V (2013). Use of the polymerase chain reaction for the diagnosis of asymptomatic *Leishmania* infection in a visceral leishmaniasis-endemic area. Rev Inst Med Trop Sao Paulo.

[98] Añez N, Rojas A, Vargas-Diaz E, Medina V, Crisante G, Yepez JY (2012). Estudio epidemiológico sobre leishmaniasis visceral en la región semiárida del occidente de Venezuela con especial referencia a la detección de infecciones inaparentes. Bol Malariol Salud Ambient.

[99] dos Santos Marques LH, Gomes LI, da Rocha IC, da Silva TA, Oliveira E, Morais MH (2012). Low parasite load estimated by qPCR in a cohort of children living in urban area endemic for visceral leishmaniasis in Brazil. PLoS Negl Trop Dis.

[100] Lima ID, Queiroz JW, Lacerda HG, Queiroz PV, Pontes NN, Barbosa JD (2012). *Leishmania infantum chagasi* in northeastern Brazil: asymptomatic infection at the urban perimeter. Am J Trop Med Hyg.

[101] Carneiro M, Castro E, Goncalves A, Lambertucci JR, Antunes C (2011). Visceral leishmaniasis: challenges in identifying subclinical *Leishmania* infection. Drug Dev Res.

[102] Silva LA, Romero HD, Nogueira Nascentes GA, Costa RT, Rodrigues V, Prata A (2011). Antileishmania immunological tests for asymptomatic subjects living in a visceral leishmaniasis-endemic area in Brazil. Am J Trop Med Hyg.

[103] Crescente JA, Silveira FT, Lainson R, Gomes CM, Laurenti MD, Corbett CE (2009). A cross-sectional study on the clinical and immunological spectrum of human *Leishmania* (L.) *infantum**chagasi* infection in the Brazilian Amazon region. Trans R Soc Trop Med Hyg.

[104] Romero HD, Silva Lde A, Silva-Vergara ML, Rodrigues V, Costa RT, Guimaraes SF (2009). Comparative study of serologic tests for the diagnosis of asymptomatic visceral leishmaniasis in an endemic area. Am J Trop Med Hyg.

[105] de Gouvea VL, de Assis TS, Orsini M, da Silva AR, de Souza GF, Caligiorne R (2008). Combined diagnostic methods identify a remarkable proportion of asymptomatic *Leishmania* (*Leishmania*) *chagasi* carriers who present modulated cytokine profiles. Trans R Soc Trop Med Hyg.

[106] de Oliveira A, Paniago AM, Sanches MA, Dorval ME, Oshiro E, Leal C, de Paula FH, Pereira LG, da Cunha R, Bóia M (2008). Asymptomatic infection in family contacts of patients with human visceral leishmaniasis in Três Lagoas, Mato Grosso do Sul State, Brazil. Cad Saúde Pública.

[107] Brandão Nascimento M, de Barros Bezerra F, Neto A, da Silva L, Bezerra J, de Castro Viana GM (2006). Estudo comparativo de anticorpos IgG e IgE antileishmania como marcadores de infecção e doença em indivíduos de área endêmica de leishmaniose visceral, em São Luis, MA. Rev Soc Bras Med Trop.

[108] Moreno EC, Melo MN, Lambertucci JR, Serufo JC, Andrade AS, Antunes CM (2006). Diagnosing human asymptomatic visceral leishmaniasis in an urban area of the State of Minas Gerais, using serological and molecular biology techniques. Rev Soc Bras Med Trop.

[109] Brandão Nascimento M, Souza E, da Silva L, da Cunha Leal L, Cantanhede C, Bezerra G, de Castro Viana GC (2005). Prevalência de infecção por *Leishmania chagasi* utilizando os métodos de ELISA (rK39 e CRUDE) e intradermorreação de Montenegro em área endêmica do Maranhão, Brasil. Cad Saúde Pública.

[110] Braz RF, Nascimento ET, Martins DR, Wilson ME, Pearson RD, Reed SG (2002). The sensitivity and specificity of *Leishmania chagasi* recombinant K39 antigen in the diagnosis of American visceral leishmaniasis and in differentiating active from subclinical infection. Am J Trop Med Hyg.

[111] Caldas AJS, Silva DR, Pereira CC, Nunes PM, Silva BP, Silva AA, Barral A, Costa JM (2001). *Leishmania* (*Leishmania*) *chagasi* infection in children from an endemic area of visceral leishmaniasis in the São Luís Island-MA, Brazil. Rev Soc Bras Med Trop.

[112] Corredor AA, Agudelo CA, Bueno M, Lopez MC, Caceres E, Reyes P, Duque S, Gualdron LE, Santacruz MM (1999). Prevalence of *Trypanosoma cruzi* and *Leishmania chagasi* infection and risk factors in a Colombian indigenous population. Rev Inst Med Trop Sao Paulo.

[113] Guarín N, Palma G, Pirmez C, Valderrama L, Tovar R, Saravia N (2006). Comparative immunohistological analysis of the Montenegro skin test reaction in asymptomatic infection and in acute and chronic cutaneous leishmaniasis. Biomedica.

[114] Arraes SM, Marini MT, Martello D, Silveira TG, Lonardoni MV, Nanni MR (2008). Serological investigation of subclinical cutaneous leishmaniasis cases following an outbreak in an endemic area. Rev Soc Bras Med Trop.

[115] Best I, Privat-Maldonado A, Cruz M, Zimic M, Bras-Goncalves R, Lemesre JL (2018). IFN-gamma response is associated to time exposure among asymptomatic immune responders that visited American Tegumentary Leishmaniasis endemic areas in Peru. Front Cell Infect Microbiol.

[116] Guedes DL, Justo AM, Barbosa Junior WL, Silva EDD, Aquino SR, Lima Junior M (2021). Asymptomatic *Leishmania* infection in HIV-positive outpatients on antiretroviral therapy in Pernambuco. Brazil PLoS Negl Trop Dis.

[117] Cunha MA, Celeste BJ, Kesper N, Fugimori M, Lago MM, Ibanes AS (2020). Frequency of *Leishmania* spp. infection among HIV-infected patients living in an urban area in Brazil: a cross-sectional study. BMC Infect Dis.

[118] Orsini M, Canela JR, Disch J, Maciel F, Greco D, Toledo A (2012). High frequency of asymptomatic *Leishmania* spp. infection among HIV-infected patients living in endemic areas for visceral leishmaniasis in Brazil. Trans R Soc Trop Med Hyg.

[119] Clemente WT, Rabello A, Faria LC, Peruhype-Magalhaes V, Gomes LI, da Silva TA (2014). High prevalence of asymptomatic *Leishmania* spp. infection among liver transplant recipients and donors from an endemic area of Brazil. Am J Transplant..

[120] van Griensven J, Carrillo E, Lopez-Velez R, Lynen L, Moreno J (2014). Leishmaniasis in immunosuppressed individuals. Clin Microbiol Infect.

[121] Molina R, Gradoni L, Alvar J (2003). HIV and the transmission of *Leishmania*. Ann Trop Med Parasitol.

[122] Srivastava P, Dayama A, Mehrotra S, Sundar S (2011). Diagnosis of visceral leishmaniasis. Trans R Soc Trop Med Hyg.

[123] WHO (2020). Ending the neglected to attain the sustainable development goals. A road map for neglected tropical diseases 2021–2030.

[124] Bezuneh A, Mukhtar M, Abdoun A, Teferi T, Takele Y, Diro E (2014). Comparison of point-of-care tests for the rapid diagnosis of visceral leishmaniasis in East African patients. Am J Trop Med Hyg.

[125] Kuhne V, Rezaei Z, Pitzinger P, Buscher P (2019). Systematic review on antigens for serodiagnosis of visceral leishmaniasis, with a focus on East Africa. PLoS Negl Trop Dis.

[126] Rosales-Chilama M, Diaz-Moreno N, Prieto MD, Giraldo-Parra L, Martinez-Valencia AJ, Gomez MA (2020). Comparative assessment of DNA targets and amplification methods for *Leishmania* (*Viannia*) detection in human samples. Am J Trop Med Hyg.

