# Supplementary material for: Identification of asymptomatic Leishmania infections: a scoping review
Source: Parasit Vectors. 2022 Jan 5;15:5. doi: 10.1186/s13071-021-05129-y (PMC8727076; doi:10.1186/s13071-021-05129-y)
Supplement: Supplementary file 1 — Additional file 1: Table S1. List of countries considered endemic. [file 13071_2021_5129_MOESM1_ESM.docx]

# Supplementary information

Additional table S1. List of countries considered endemic

| **Location** | **Status of endemicity VL** | **Status of endemicity CL** |
| --- | --- | --- |
| **Afghanistan** | Endemic | Endemic |
| **Albania** | Endemic | Endemic |
| **Algeria** | Endemic | Endemic |
| **Angola** | Previously reported cases | No autochthonous cases reported |
| **Argentina** | Endemic | Endemic |
| **Armenia** | Endemic | Endemic |
| **Azerbaijan** | Endemic | Endemic |
| **Bangladesh** | Endemic | No autochthonous cases reported |
| **Belize** | No autochthonous cases reported | Endemic |
| **Bhutan** | Endemic | Endemic |
| **Bolivia** | Endemic | Endemic |
| **Bosnia and Herzegovina** | Endemic | Endemic |
| **Brazil** | Endemic | Endemic |
| **Bulgaria** | Endemic | Endemic |
| **Burkina Faso** | No autochthonous cases reported | Endemic |
| **Cameroon** | Endemic | Endemic |
| **Central African Republic** | Previously reported cases | Previously reported cases |
| **Chad** | Endemic | Endemic |
| **China** | Endemic | Endemic |
| **Colombia** | Endemic | Endemic |
| **Costa Rica** | Endemic | Endemic |
| **Côte d'Ivoire** | Endemic | Endemic |
| **Croatia** | Endemic | Endemic |
| **Cyprus** | Endemic | Endemic |
| **Democratic Republic of the Congo** | Endemic | Endemic |
| **Dijbouti** | Endemic | Endemic |
| **Dominican Republic** | No autochthonous cases reported | Endemic |
| **Ecuador** | No autochthonous cases reported | Endemic |
| **Egypt** | Endemic | Endemic |
| **El Salvador** | Endemic | Endemic |
| **Eritrea** | Endemic | Endemic |
| **Ethiopia** | Endemic | Endemic |
| **France** | Endemic | Endemic |
| **Gambia** | Previously reported cases | Previously reported cases |
| **Georgia** | Endemic | Endemic |
| **Ghana** | No autochthonous cases reported | Endemic |
| **Greece** | Endemic | Endemic |
| **Guatemala** | Endemic | Endemic |
| **Guinea** | No autochthonous cases reported | Endemic |
| **Guinea-Bissau** | No autochthonous cases reported | Endemic |
| **Guyana** | No autochthonous cases reported | Endemic |
| **Honduras** | Endemic | Endemic |
| **India** | Endemic | Endemic |
| **Iran** | Endemic | Endemic |
| **Iraq** | Endemic | Endemic |
| **Israel** | Endemic | Endemic |
| **Italy** | Endemic | Endemic |
| **Jordan** | Endemic | Endemic |
| **Kazakhstan** | Endemic | Endemic |
| **Kenya** | Endemic | Endemic |
| **Kuwait** | No autochthonous cases reported | Endemic |
| **Kyrgyztan** | Endemic | Endemic |
| **Lebanon** | Endemic | Endemic |
| **Libya** | Endemic | Endemic |
| **Malawi** | No autochthonous cases reported | Endemic |
| **Mali** | No autochthonous cases reported | Endemic |
| **Malta** | Endemic | Endemic |
| **Mauritania** | Endemic | Endemic |
| **Mexico** | Endemic | Endemic |
| **Monaco** | Endemic | Endemic |
| **Montenegro** | Endemic | Endemic |
| **Morocco** | Endemic | Endemic |
| **Namibia** | No autochthonous cases reported | Endemic |
| **Nepal** | Endemic | Endemic |
| **Nicaragua** | Endemic | Endemic |
| **Niger** | Endemic | Endemic |
| **Nigeria** | Previously reported cases | Endemic |
| **Oman** | Endemic | Endemic |
| **Pakistan** | Endemic | Endemic |
| **Panama** | No autochthonous cases reported | Endemic |
| **Paraguay** | Endemic | Endemic |
| **Peru** | No autochthonous cases reported | Endemic |
| **Portugal** | Endemic | Endemic |
| **The former Yugoslav Republic of Macedonia** | Endemic | Endemic |
| **Romania** | Endemic | No autochthonous cases reported |
| **Saudi Arabia** | Endemic | Endemic |
| **Senegal** | Endemic | Endemic |
| **Serbia** | Previously reported cases | No autochthonous cases reported |
| **Slovenia** | Endemic | Endemic |
| **Somalia** | Endemic | No autochthonous cases reported |
| **South Sudan** | Endemic | No autochthonous cases reported |
| **Spain** | Endemic | Endemic |
| **Sri Lanka** | Endemic | Endemic |
| **Sudan** | Endemic | Endemic |
| **Suriname** | No autochthonous cases reported | Endemic |
| **Syrian Arab Republic** | Endemic | Endemic |
| **Tajikistan** | Endemic | Endemic |
| **Thailand** | Endemic | Endemic |
| **Tunisia** | Endemic | Endemic |
| **Turkey** | Endemic | Endemic |
| **Turkmenistan** | Endemic | Endemic |
| **Uganda** | Endemic | No autochthonous cases reported |
| **Ukraine** | Endemic | Previously reported cases |
| **United States of America** | No autochthonous cases reported | Endemic |
| **Uruguay** | Endemic | No autochthonous cases reported |
| **Uzbekistan** | Endemic | Endemic |
| **Venezuela** | Endemic | Endemic |
| **Yemen** | Endemic | Endemic |
| **Zambia** | Endemic | No autochthonous cases reported |
