# Supplementary material for: Identification of asymptomatic Leishmania infections: a scoping review
Source: Parasit Vectors. 2022 Jan 5;15:5. doi: 10.1186/s13071-021-05129-y (PMC8727076; doi:10.1186/s13071-021-05129-y)
Supplement: Supplementary file 2 — Additional file 2: Table S2. Search syntax for PubMed. [file 13071_2021_5129_MOESM2_ESM.docx]

**Supplementary Table 2.** Search syntax for PubMed

| **Original search syntax**  ((Leishmania[MeSH Terms]) OR (leishmania*)) AND (((asymptomatic*) OR (carrier) OR (blood donor) OR (subclinical))) |
| --- |
| **Complete search syntax**  ("leishmania"[MeSH Terms] OR "leishmania*"[All Fields]) AND ("asymptomatic*"[All Fields] OR ("carrier state"[MeSH Terms] OR ("carrier"[All Fields] AND "state"[All Fields]) OR "carrier state"[All Fields] OR "carrier"[All Fields] OR "carrier s"[All Fields] OR "heterozygote"[MeSH Terms] OR "heterozygote"[All Fields] OR "carriers"[All Fields]) OR ("blood donors"[MeSH Terms] OR ("blood"[All Fields] AND "donors"[All Fields]) OR "blood donors"[All Fields] OR ("blood"[All Fields] AND "donor"[All Fields]) OR "blood donor"[All Fields]) OR ("subclinic"[All Fields] OR "subclinical"[All Fields] OR "subclinically"[All Fields] OR "subclinicals"[All Fields]) |
| **Translations**  **leishmania[MeSH Terms]:** "leishmania"[MeSH Terms]  **carrier:** "carrier state"[MeSH Terms] OR ("carrier"[All Fields] AND "state"[All Fields]) OR "carrier state"[All Fields] OR "carrier"[All Fields] OR "carrier's"[All Fields] OR "heterozygote"[MeSH Terms] OR "heterozygote"[All Fields] OR "carriers"[All Fields]  **blood donor:** "blood donors"[MeSH Terms] OR ("blood"[All Fields] AND "donors"[All Fields]) OR "blood donors"[All Fields] OR ("blood"[All Fields] AND "donor"[All Fields]) OR "blood donor"[All Fields]  **subclinical:** "subclinic"[All Fields] OR "subclinical"[All Fields] OR "subclinically"[All Fields] OR "subclinicals"[All Fields] |
